# Understanding how informal dementia caregiver networks are assessed in the literature: results from a scoping review

**DOI:** 10.3389/frdem.2026.1824829

**Published:** 2026-06-12

**Authors:** Noelle E. Carlozzi, Wendy L. Lombard, Madison Fansher, Jacqueline L. Freeman, Emily Capellari, Christopher M. Graves, Jennifer A. Miner, Benjamin M. Hampstead, Jin-Shei Lai, Amanda N. Leggett

**Affiliations:** 1 Center for Clinical Outcomes Development and Application, Department of Physical Medicine and Rehabilitation, University of Michigan, Ann Arbor, MI, United States; 2 Department of Surgery, University of Michigan, Ann Arbor, MI, United States; 3 Institute for Social Research, University of Michigan, Ann Arbor, MI, United States; 4 Taubman Health Sciences Library, University of Michigan, Ann Arbor, MI, United States; 5 Department of Psychiatry, University of Michigan, Ann Arbor, MI, United States; 6 Department of Neurology, University of Michigan, Ann Arbor, MI, United States; 7 Department of Medical Social Sciences, Northwestern University, Chicago, IL, United States; 8 Institute of Gerontology, Wayne State University, Detroit, MI, United States; 9 Department of Psychology, Wayne State University, Detroit, MI, United States

**Keywords:** activities of daily living, Alzheimer’s disease, caregiver networks, caregivers, patient care team

## Abstract

**Objectives:**

Alzheimer’s disease and related dementias currently affect millions of Americans, with increasing prevalence expected as the population ages. Care for individuals with dementia is typically provided by informal caregiver networks encompassing family, friends, and nontraditional care partners. Despite societal changes in caregiving roles and family structure, there is limited consensus on how caregiver networks are conceptualized and measured in research literature. The purpose of this analysis was to systematically map existing measurement approaches used to capture and characterize informal dementia caregiver networks, and to identify common data elements and validated instruments within the literature.

**Methods:**

We conducted a rigorous literature review guided by the JBI Manual for Evidence Synthesis for conducting scoping reviews, the Preferred Reporting Items for Systematic reviews and Metal-Analyses extension for Scoping Reviews (PRISMA-ScR). The protocol was registered on OSF (DOI: 10.17605/OSF. IO/2XGSR). Inclusion criteria comprised peer-reviewed, quantitative, English-language articles that defined or measured caregiver networks, with no geographic or date restrictions. Independent raters performed title, abstract, and full-text screening, supplemented by artificial intelligence (AI)-supported data extraction and latent content analysis to identify measurement approaches and data elements.

**Results:**

Out of 14,625 initial references, 197 studies were included that assessed caregiver networks for individuals with dementia. Only 19 studies utilized validated instruments, while the remaining 177 (89%) relied on study-specific measures. Thirteen validated tools were identified, although most were used only once or twice. Latent content analysis revealed seven recurring data elements in network characterization: (1) availability of another caregiver (45.2%), (2) amount of help provided (39.1%), (3) number of caregivers (23.4%), (4) type of activities assisted with (16.2%), (5) relationships of helpers (13.7%), (6) satisfaction with support (12.7%), and (7) demographic details of caregivers (2.5%). Most studies assessed just one or two network attributes, with marked variability and limited adoption of standardized measures.

**Conclusion:**

There is substantial heterogeneity and inconsistency in how informal caregiver networks are assessed in dementia research, and there is no consensus approach in the literature. As caregiving networks diversify in response to changing demographics and family structures, robust, inclusive assessment instruments are critically needed. Future research should prioritize developing and validating standardized measures to better capture the complexities of modern informal dementia caregiver networks, thereby enhancing policy, support, and interventions for these populations.

**Systematic review registration:**

OSF Registration found at https://osf.io/2xgsr/overview.

## Introduction

Alzheimer’s disease currently afflicts over seven million Americans aged 65 and older, and this number is expected to grow to 12.7 million by 2050 (Alzheimer's Association, 2025; Dhana et al., 2023; Hebert et al., 2003; Weuve et al., 2015). One in nine older adults in the U. S. have Alzheimer’s disease; two thirds of these are women, with the disease disproportionately affecting racial and ethnic minority individuals (Alzheimer's Association, 2025). Alzheimer’s disease and related dementias cause loss of cognitive function and behavioral changes that interfere with one’s ability to complete everyday household and self-care tasks and participate in social activities (Safiri et al., 2024). Eventually, the individual may become unable to perform even simple tasks and need increasingly complex levels of care and support from others. Nearly 12 million Americans provide unpaid care for someone with dementia, providing an estimated 19.2 billion hours of informal (i.e., unpaid) assistance in 2024, a contribution to the nation valued at $413.5 billion (Alzheimer's Association, 2025).

Informal dementia caregiving is typically provided through a network individuals, primarily spouses and adult biological children (Wu et al., 2024). The availability of close family members to provide unpaid care has been a long-held expectation in U. S. society that is embedded in the country’s long-term care policy framework. But societal changes in family structures (e.g., fewer marriages, lower fertility rate, rapidly growing elderly population) have increased the likelihood that unpaid care will be provided by a broader network of informal caregivers who may or may not be related to the person with dementia (Wu et al., 2024). These include unmarried domestic partners, “families of origin” (i.e., siblings and other blood relatives), in-laws, step-kin, step- and adopted children, and “families of choice” (e.g., personal communities of people such as friends, neighbors, church members, and other people with whom individuals share a kin-like relationship but are not connected by biological or legal ties).

Research has shown that informal dementia caregiver networks, relative to non-dementia care networks, include more caregivers (78% of the time), incorporate more task sharing (including medical assistance, mobility assistance, self-care assistance), and rely more on caregivers who help in multiple task domains (as opposed to specializing in one task domain, such as only helping with transportation) (Spillman et al., 2020a, 2020b). Demographic changes within family structures also raise key questions about who individuals consider to be “family” and what roles those people play in caring for individuals with dementia (Patterson et al., 2025; Wu et al., 2024). Characterizing these caregiver networks is important for understanding the experiences and challenges of the caregiver, care recipient, and care team dynamics.

While a large body of literature focuses on evaluating health-related quality of life for individual caregivers,(Aneshensel, 1995; Cuijpers, 2005; Dura et al., 1991; Fonareva and Oken, 2014; Hinrichsen and Zweig, 1994; Mahoney et al., 2005; Mausbach et al., 2013; Perkins et al., 2012; Pinquart et al., 2006; Pinquart and Sörensen, 2006; Schulz and Beach, 1999; von Känel et al., 2006; Zarit, 2008) few studies have sought to examine extended informal caregiver networks. Broad societal changes in demographic, marriage, fertility, and social structures; as well as an increasing population of older adults with dementia, are changing how care is provided to individuals with dementia. Despite these factors, the current literature reveals notable inconsistencies in how caregiving networks are conceptualized and measured. Studies frequently use varying definitions, sometimes conflating “social networks” used for general social support with “caregiving networks” specifically composed of individuals actively providing care (Spillman et al., 2020a, 2020b; Wu et al., 2024). This lack of clarity can obscure important distinctions regarding the roles, relationships, and task distribution among network members. For example, some research relies on informal interviews or caregiver self-report questionnaires, while others employ social network analysis, egocentric mapping, or administrative data to capture care connections. These disparate approaches result in a wide range of findings regarding caregiving structures, hindering direct comparisons and synthesis across studies. Consequently, there is a need for standardized, validated measures that delineate who is counted as a caregiver, the nature of their involvement, and the structure of caregiving networks distinct from broader social support systems. Achieving greater clarity in measurement and conceptualization will enable a more robust understanding of the complexities and challenges within dementia caregiver networks.

To address this need, we conducted a scoping review of the literature on dementia caregiving to identify existing measurement approaches for capturing and characterizing an individual’s care team or network. The protocol was developed in accordance with guidance from the JBI Manual for Evidence Synthesis for scoping reviews(Aromataris et al., 2024) and the review is reported using the Preferred Reporting Items for Systematic Reviews and Meta-Analyses guidelines extension for Scoping Reviews^*^ (PRISMA-ScR) (Tricco et al., 2018) and was pre-registered at: https://osf.io/2xgsr/overview. Detailed study procedures were peer-reviewed and published previously (Carlozzi et al., 2025). We initially identified over 9,000 studies to review; after removing duplicates and irrelevant studies, our final pool of articles was 5,273. These studies included caregiver and patient populations across diverse racial, ethnic, and socioeconomic groups within and outside of the United States.

## Materials and methods

Searches were designed and run by a health sciences librarian (JF) in the following databases from inception to December 15, 2023, to identify relevant articles describing items, tools, measures, or approaches to defining non-traditional care partners of people with dementia: Ovid MEDLINE(R) ALL, PsycInfo (EBSCOhost), AgeLine (EBSCOhost), and CINAHL Complete (EBSCOhost). Each search used controlled vocabulary where possible combined with keywords, derivatives, and synonyms for select dementia-related clinical diagnoses; informal caregivers, caregivers, relative types, or family structure; and measures (Supplemental File). No limits were applied to the search. Search terms were initially generated through a collaborative process with other review team members to generate a draft search for Ovid MEDLINE. A selection of articles was reviewed for relevance and refinement of the draft search strategy. A second health sciences librarian (EC) peer-reviewed the refined strategy according to PRESS guidelines (McGowan et al., 2016). The refined Ovid MEDLINE search was translated to the other databases. Citations were deduplicated on import to Covidence and through manual identification. Following guidance cited from Booth 2016 in the Cochrane Handbook (Lefebvre et al., 2025) for when to stop searching in evidence synthesis reviews of qualitative data, authors of this review determined that new information was no longer being identified and a search update was not warranted.

### Inclusion and exclusion criteria

To be included, articles had to: (1) be published or available in English; (2) be peer-reviewed; (3) describe completed, quantitative research in a dementia caregiving sample; and (4) contain *either* a caregiver definition, *or* surveys/tools/items that describe the caregiver network (this was the focus of the current inquiry), *or* contain caregiver-specific measures/tools/items. We excluded articles that: (1) did not include data; (2) had a restricted sample size (*N* < 20); (3) focused solely on professional, paid caregivers; (4) only included qualitative data; (5) were part of the grey literature (e.g., dissertations, published abstracts); or (6) were review articles, case studies, response letters, research reports, graduate theses, and published study protocols.

### Screening and extraction procedure

We initially identified over 14,000 studies to review; after removing duplicates (by Covidence systematic review software (Veritas Health Innovation, 2026) and through manual identification) and irrelevant studies, our final pool of articles was 5,273. Title and abstract screening was conducted by two independent raters; discrepancies were reconciled, and a third rater was introduced if consensus could not be achieved. Next, the full text for the included articles was retrieved and reviewed by two independent raters for inclusion; discrepancies were reconciled, and a third rater was introduced if consensus could not be achieved. Data extraction included author details, year of publication, sample size, caregiver population(s), caregiver definition (not included in the current inquiry), assessment tools used to capture details about the caregiver network (the focus of the current inquiry), and other outcomes measures that were evaluated (not included in the current inquiry). Data extraction was supported by the artificial intelligence (AI)-based software “U-M Maizey.” U-M Maizey is part of the University of Michigan’s suite of generative AI tools maintained by the University and is available to all faculty, staff, and students. The AI-based extractions were reviewed by an independent, human rater, and any identified discrepancies were reviewed by a second independent rater. Following this process, we conducted an additional round of review that involved excluding all references that solely focused on the caregiver’s social support network (i.e., without specific reference to actual support related to the caregiver role). Finally, content analysis (Catanzaro, 1988; Graneheim and Lundman, 2004; Hsieh and Shannon, 2005; Miles et al., 2014) was implemented to identify approaches and measures for capturing and characterizing the caregiver network.

## Results

**Figure 1:**
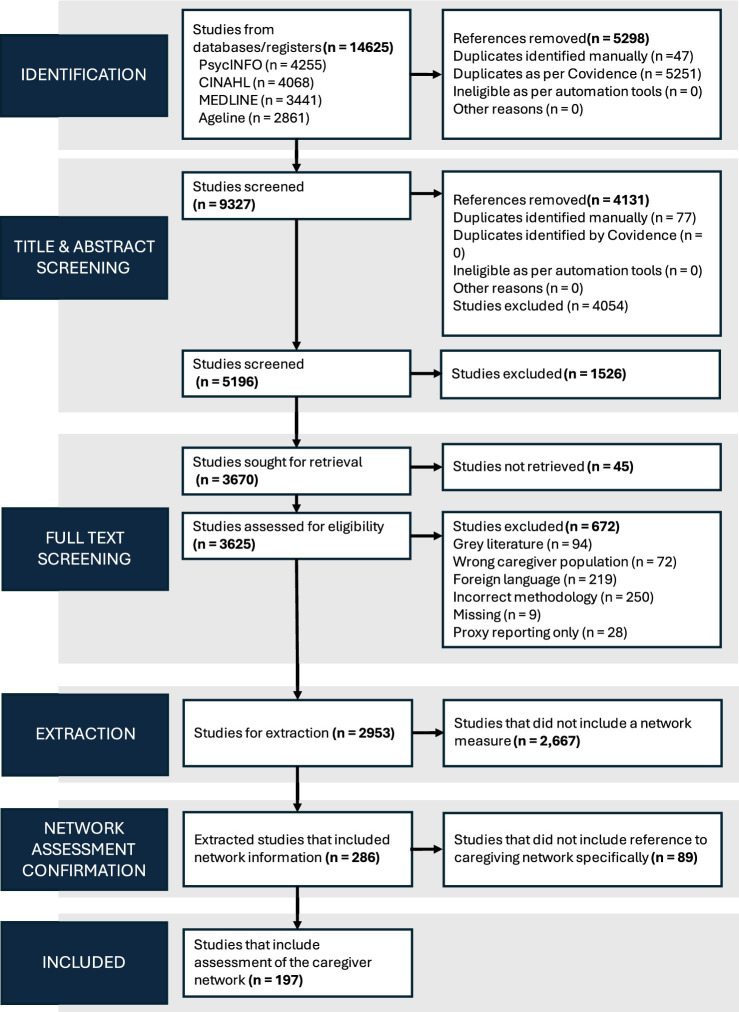
PRISMA flow diagram of scoping review to identify existing measurement approaches characterizing dementia care networks.

The initial search identified 14,625 articles. After duplicates and irrelevant studies were removed, 9,327 articles underwent initial title and abstract screening, 5,196 underwent a second round of title and abstract screening, 3,625 studies were advanced to full text screening, and 2,953 underwent extraction. Of these 3,625, 286 included studies that referenced the assessment of a social network (which initially included both social networks and caregiving networks). Of these 286 articles, 197 provided information specifically about the caregiver network. A flowchart for this process and the exclusion details are provided in Figure 1. Of these 197 studies, 19 included validated instruments and 177 included study-specific measures (please note that of the total 197 studies, n = 6 included both validated instruments and study-specific measures). Citations for the 197 articles that were the focus of this analysis are included in the Appendix.

Table 1 provides a summary of the different validated assessments that were used. Of the 19 studies that included validated instruments or approaches, 13 different measures were utilized. Nine of these measures were used only once, two were used twice (Family Conflict Scale; Pearlin et al., 1990) and the CarerQOL-7D Scale (Brouwer et al., 2006b), and two were used three times (the Resource Utilization in Dementia RUD; Wimo et al., 1998) or RUD-Lite (Wimo et al., 2013) and the Inventory of Socially Supportive Behavior (ISSB) (Barrera et al., 1981). In general, the psychometric properties of these tools were adequate to good, and almost all studies utilized individual items as opposed to the validated scores, making the actual reliability and validity of their usage unknown.

**Table 1:** Validated assessments used in the literature to evaluate components of the caregiver network.

Validated assessment	Number of studies using it	Primary citation	Brief description	Summary of psychometric properties	Domain assessed
Inventory of Socially Supportive Behavior (ISSB)	3	Barrera et al. (1981)	Forty items assessing instrumental assistance and emotional relationships (includes a single network item that assesses looking after a family member while you were away)	Internal consistency ranges from 0.79 to 0.94; test–retest ICC = 0.88; construct, convergent, and predictive validity supported (Barrera et al., 1981; Stokes and Wilson, 1984)	Captures: (1) whether or not another person is available to provide care for the person with dementia
Resource Utilization in Dementia (RUD or RUD-Lite)	3	Wimo et al. (1998, 2013)	~25 items for the RUD and ~8 items for the RUD-Lite that assess resource usage among patients with dementia (includes time spent on formal and informal care by caregivers--reported as number of hours per day during a typical care day in the past month)	Reliability estimates of time ICCs range from 0.75 to 0.93 (Wimo et al., 2010; Wimo and Nordberg, 2007)	Captures: (1) the amount of help each caregiver provides; and (2) the type of assistance provided
CarerQoL-7D	2	Brouwer et al. (2006b)	Seven items that assess support with carrying out care tasks includes a single network item: I have [no, some, a lot of]…support with carrying out my care tasks, when I need it (e.g., from family, friends, neighbors, acquaintances).	Internal consistency ranges from 0.65 to 0.67; ICCs range from 0.55 to 0.94; convergent, clinical, and discriminant validity supported (Brouwer et al., 2006a; Cejalvo et al., 2025; Hoefman et al., 2013; Hoefman et al., 2011a; Hoefman et al., 2011b; Lutomski et al., 2015; McCaffrey et al., 2020; McLoughlin et al., 2020; Voormolen et al., 2021)	Captures: (1) whether or not another person is available to provide care for the person with dementia
Family conflict	2	Pearlin et al. (1990)	Twelve items that assess caregiver conflict with the family regarding the care of the relative, expressive support, the caregiver’s feelings of being understood, feeling close to people, having people they can trust and confide in, and people that they want to be with and who make them feel good. (includes 1 item that assesses satisfaction with informal help)	Internal consistency ranged from 0.80 to 0.87 (Pearlin et al., 1990)	Captures: (1) the satisfaction that the caregiver has with the support provided by other members of the network
Arizona Social Support Interview Schedule (ASSIS)	1	Barrera (1980)	Perceptions of need for, satisfaction with, and availability of social support in the past month; measures the number of people (e.g., family, friends, neighbors, clergy, professionals) perceived as available and the number who actually provide support in each of six domains.	Internal consistency = 0.78 test–retest ICCs range from 0.33 to 0.88; convergent validity supported (Barrera, 1980; Heitzmann and Kaplan, 1988)	Captures: (1) the number of people that provide care for the person living with dementia
Caregiving support	1	Hyodo et al. (2003)	Ten items that assess informal and formal support (includes the number of family members, friends, and neighbors providing emotional (two items), instrumental (two items), and informational (one item) support, as well as satisfaction).	Unable to evaluate; published article only available in Japanese	Captures: (1) the number of people that provide care for the person living with dementia; and (2) the satisfaction that the caregiver has with the support provided by other members of the network
Duke Social Support Index	1	George and Gwyther (1986)	Ten items that assess caregivers’ satisfaction with their relationships (includes a single item concerning caregivers’ desires for more help from family and friends)	Internal consistency ranges from 0.64 to 0.90; split-half reliability = 0.92; construct validity generally supported; convergent and discriminant validity supported (George and Gwyther, 1986; Jia and Zhang, 2012; Mao et al., 2015; Pan et al., 2020)	Captures: (1) the satisfaction that the caregiver has with the support provided by other members of the network
Family Caregiving Inventory	1	Archbold and Stewart (1996)	Three items that assess the amount of available help from others (e.g., relatives, friends and neighbors, and paid assistants)	Internal consistency ranges from 0.88 to 0.94; test–retest ICC = 0.92; construct validity supported (Archbold and Stewart, 1996; Carter et al., 1998; Hudson and Hayman-White, 2006; Pucciarelli et al., 2014)	Captures: (1) the amount of help each caregiver provides
Impact of Family Caregiving Questionnaire (IFCQ)	1	Fast et al. (2019)	Five items that assess informal support (includes the number of family members and friends who provided direct help (e.g., feeding, bathing) in caring for the dependent; number of people who provided indirect help (e.g., shopping, housework); the frequency of help received; perceived quality of this assistance; and the overall satisfaction with the help received)	Internal consistency = 0.97 (Fast et al., 2019)	Captures: (1) the number of people that provide care for the person living with dementia; (2) the type of assistance provided; and (3) the satisfaction that the caregiver has with the support provided by other members of the network
MacArthur social support scale	1	Gurung et al. (2003)	~Six items that assess emotional support, instrumental support, and negative interaction involving conflict or excessive demands (includes two items on instrumental support: “How often can you count on your (spouse and children/relatives and friends) to help with daily tasks like shopping or help you with household tasks?” and “How often does/do your (spouse and children/relatives and friends) give you advice or information about medical, financial, or family problems?”)	Internal consistency = 0.78; construct validity supported (Au et al., 2009)	Captures: (1) the amount of help each caregiver provides
Social support	1	Dimond et al. (1987)	Thirteen items that assess network size, strength of ties, and the extent to which network members know each other, and the interactional quality of the network	Not reported	Captures: (1) the number of people that provide care for the person living with dementia; and (2) the satisfaction that the caregiver has with the support provided by other members of the network
Social support appraisals (SS-A) Scale	1	Vaux et al. (1986)	Twenty-three items that assess the degree to which one feels loved and respected (includes an item about if participant received paid help and hours of paid help received, whether the participant received help from other family members and hours of help received).	Internal consistency reliability, and convergent validity supported(O'Reilly, 1995)	Captures: (1) whether or not another person is available to provide care for the person with dementia; (2) who provides help; and (3) the amount of help received
A Procedure for Surveying Personal Networks	1	McCallister and Fischer (1978)	An approach for gathering information about whether or not other people are available to help with household tasks, who these individuals are, as well as their relationship (includes questions about the number of people providing some form of support to the demented person)	Not reported	Captures: (1) the number of people that provide care for the person living with dementia

We next conducted a latent content analysis of the extracted study-specific measures (please note that study-specific measures include standardized data elements that are used routinely in cohort studies) and identified seven different data elements that were used to describe the caregiver network. Specifically, these data elements included: (1) a simple assessment of whether or not another person is available to provide care for the person with dementia (most commonly assessed as a dichotomous variable: yes/no); (2) network size (the number of people who provide care for the person living with dementia; most often captured as a continuous variable, but occasionally assessed as a categorical variable that is capped at anywhere from 5 to 12 additional caregivers); (3) an indication of who provides help (typically captured as the relationship of the caregiver to the care recipient, or as paid and unpaid helpers); (4) the amount of help each caregiver provides (most commonly captured as an estimated number of hours); (5) the type of assistance provided by each caregiver (typically capturing the different activities of daily living or instrumental activities of daily living that caregivers assist with); (6) the satisfaction that the primary caregiver has with the support provided by other members of the network; and (7) demographic details about the different members of the caregiver network (e.g., age, sex, race). See Table 2.

**Table 2:** Seven common data elements from study-specific measures of the caregiver network.

Caregiver network variable	Percentage of studies that include the data element
Is someone else available to provide care? (simple dichotomy)	45.2%
How much help do others provide?	39.1%
How many people provide care? (continuous)	23.4%
What types of activities do others help with?	16.2%
Who provides help?	13.7%
Satisfaction with caregiver help provided by others	12.7%
Demographic details for other caregivers	2.5%

Across all measures (validated and study-specific), the most commonly assessed data element was the inclusion of a dichotomous variable indicating whether or not help was available (45.2%, 89/197), this was followed by the assessment of the amount of help (i.e., the estimated number of hours of help from others; 39%; 77/197), how many people provided help (23.4%, 46/197), type of help (16.2%, 32/197), an indication of who provides help (13.7%, 27/197), satisfaction with the help/support received (12.7%, 25/197), and demographic data about the helpers (2.5%, 5/197). In addition, 58.4% (115/197) of the studies assessed a single data element (i.e., one of the seven data elements referenced above), 26.4% (52/197) assessed two data elements, and 15% (30/197) included three or more data elements.

## Discussion

**Figure 2:**
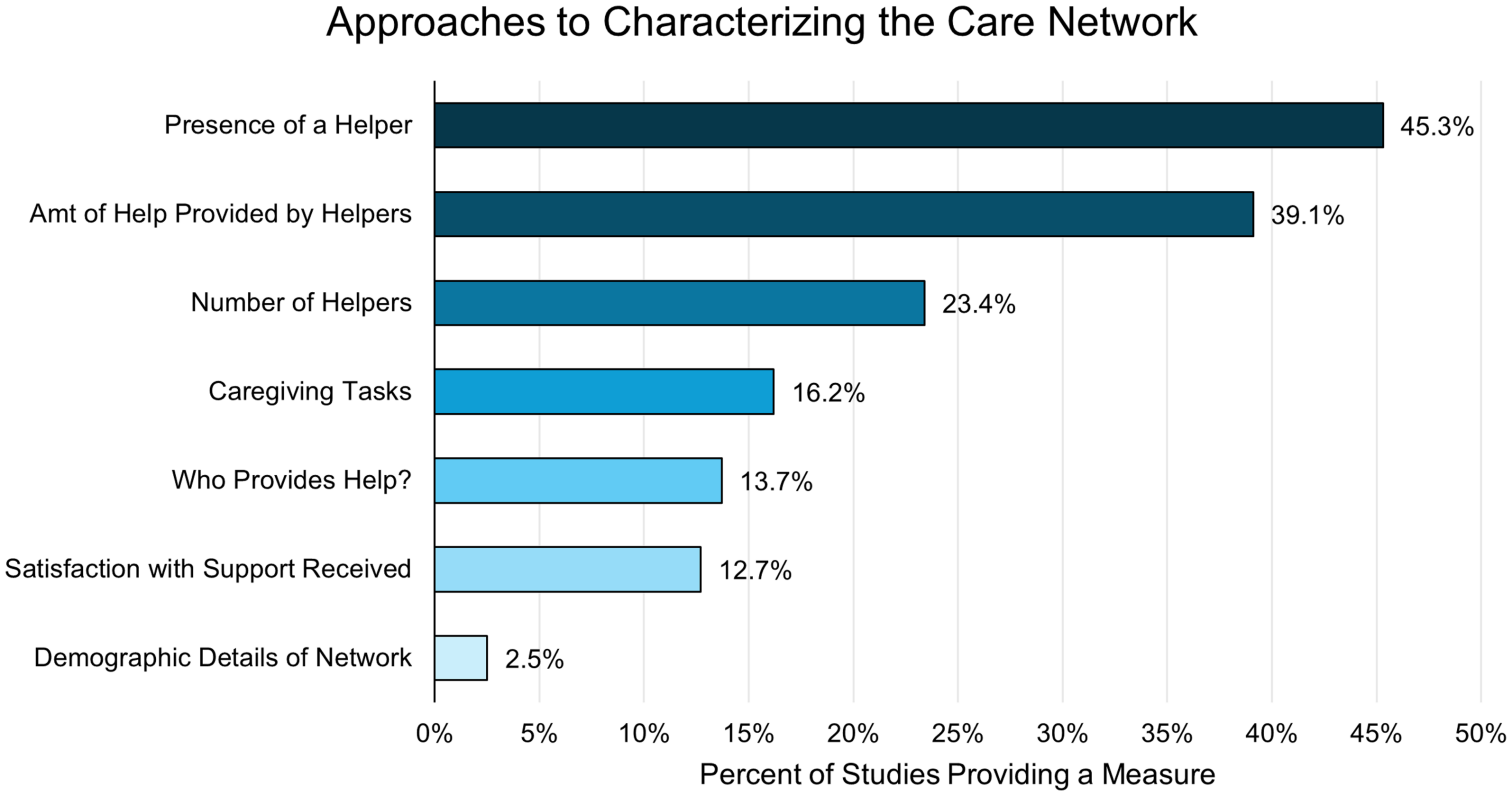
Approaches to characterizing the care network.

Taken together, the results from this scoping review indicate that most studies in the dementia literature neglect to evaluate the informal caregiver network. Among those that do, few validated measures are available and there is no consensus approach for assessing the network; the vast majority of studies ask their own study-specific questions or a subset of items from a validated measure to capture this information. Regardless, a number of specific data elements were commonly used across studies (even if they differed slightly from one another); see Figure 2. These included: (1) identifying whether or not another person is available to help provide care for the person with dementia (45.2%); (2) the number of people who provide help (23.4%); (3) an indication of who provides help (13.7%); (4) the amount of help each caregiver provides (39.1%); (5) the type of tasks that the helper assists with (16.2%); (6) the satisfaction that the caregiver respondent has with the support they receive (12.7%); and (7) demographic details about the different members of the caregiver network (e.g., age, sex, race; 2.5%). Furthermore, half of the studies simply utilized a dichotomous variable (50.4% or 58/115), 25% (29/115) asked solely about the amount of help, and 20% (23/115) assessed the total number of caregivers in the network.

Given these findings, we propose an approach to assess the informal dementia caregiver network that includes multiple network elements. Of the seven different approaches in the literature, we propose one that includes at least three different data elements: (1) identifying whether or not another person is available to help provide care for the person with dementia; (2) asking about network size [the number of people who help provide care (23.4%)], and (3) the type of tasks that each helper assists with (16.2%). Most studies of caregiver networks rely on self-reported data from a single caregiver, who may or may not be the primary caregiver and may have limited involvement or awareness of the full caregiving team. As a result, we do not recommend using standard assessments that attempt to capture information about all caregivers involved, since details such as the network size, the extent of help provided by others, their satisfaction, and demographic characteristics are often unknown or inaccessible to secondary caregivers. These data elements may be considered for inclusion when the respondent is the primary caregiver and knowledgeable about the composition of the care team.

Our findings underscore the need for more robust, inclusive assessment tools to capture the complexity of modern dementia caregiver networks. By aligning research practices with real-world caregiving scenarios, including both traditional and nontraditional caregiver networks, we can better address the diverse needs of caregivers and the individuals they support. Such efforts will be crucial in developing interventions and policies to improve caregiver health and well-being, ensure equitable resource distribution, and respond to the rapidly evolving landscape of dementia care in the United States.

### Limitations

This review has several limitations. First, we excluded qualitative literature, potentially omitting more in-depth examination of caregiver network dynamics. Furthermore, this review focused exclusively on peer-reviewed, English-language publications, which may have led to underrepresentation of relevant studies conducted in other languages. In addition, grey literature was also excluded, which may have resulted in missing relevant data in unpublished studies, conference papers, and dissertations. We also excluded other populations who have caregivers; there may be additional validated caregiver network measures that could potentially be used in dementia caregivers.

## Conclusion

This scoping review highlights significant gaps and inconsistencies in the characterization of informal caregiver networks for individuals with dementia. While various data elements are commonly assessed, most studies rely on limited, study-specific measures rather than standardized, validated instruments. As family structures and caregiving roles continue to evolve, particularly within diverse populations, it is crucial to develop and implement robust tools that capture the complexity of modern caregiver networks. Future work is underway to develop, validate, and standardize self-report measures that provide information about the caregiver network. Such advancements will better inform policy, support services, and interventions, for the diverse networks of people who provide care for people living with dementia.

## Data Availability

The authors of this publication will make the data used in this publication available to individuals in the scientific community upon request. Requests for data sharing should be emailed to PMR-CODALab@med.umich.edu or the corresponding author and reference this publication.
