# Supplementary material for: Understanding how informal dementia caregiver networks are assessed in the literature: results from a scoping review
Source: Front Dement. 2026 Jun 12;5:1824829. doi: 10.3389/frdem.2026.1824829 (PMC13303008; doi:10.3389/frdem.2026.1824829)
Supplement: Supplementary file 1 [file Supplementary_File_1.pdf]

Search strategies by database

Ovid Medline(R) ALL

1 exp Dementia/ or AIDS Dementia Complex/ or Alzheimer Disease/ or Dementia, Vascular/ or Lewy Body Disease/ or Mixed Dementias/ or Dementia, Multi-Infarct/ or CADASIL/ or Amyotrophic Lateral Sclerosis/ or Rett Syndrome/ or Prion Diseases/ or exp Frontotemporal Dementia/

2 ("Pick Disease of the brain" or Pick's disease or "dementia with Lewy Bodies" or posterior cortical atrophy or amentia* or dementia* or alzheimer* or aphasia$1 or mesulam syndrome or mesulam's syndrome or Creutzfeldt-Jakob Syndrome or creutzfeldt ja?ob disease$1 or creutzfeldt-ja?ob syndrome or subacute spongiform encephalopath* or "Diffuse neurofibrillary tangles with calcification" or kosaka shibayam disease or frontotemporal lobar degeneration$1 or ftld or ftlds or Huntington$1 disease or "akinetic rigid variant of huntington disease" or chronic progressive hereditary chorea or huntington$1 chorea or huntington chronic progressive hereditary chorea or chronic hereditary progressive chorea or hereditary chronic progressive chorea or Kluver-Bucy syndrome or kluver bucy syndrome or temporal lobectomy behavior syndrome or (binswanger* adj5 (disease or encephalopath*)) or lewy body disease or cadasil* or "cerebral arteriopathy with subcortical infarcts and leukoencephalopathy" or "cerebral autosomal dominant arteriopathy with subcortical infarcts and leukoencephalopathy" or ALS or amyotrophic lateral sclerosis or charcot disease or (gehrig* adj disease) or guam disease or ((rett or retts or rett's) adj (disorder* or syndrome*)) or (prion* adj3 (disease* or disorder*)) or transmissible spongiform encephalopath* or acquired immunodeficiency syndrome encephalopath* or human immunodeficiency virus encephalopath* or ((AIDS or HIV or subcortical or arteriosclerotic or chronic or progressive or leuko) adj4 encephalopath*) or ((HIV or AIDS) adj4 motor complex) or ((ALS or amyotrophic lateral sclerosis) adj15 Guam)).ab,ti,kf.

3 1 or 2

4 exp Family Structure/ or Caregivers/ or Child, Adopted/ or Child, Foster/ or Friends/ or Grandparents/ or Siblings/ or exp Legal Guardians/ or Household Work/

5 (("family structure*" or "reconstituted famil*" or stepfamil* or "step famil*" or "step-famil*" or "blended famil*" or "step kin" or "step-kin" or "stepkin" or stepparent$1 or "step parent$1" or "step-parent$1" or stepdaughter$1 or "step daughter$1" or "step-daughter$1" or stepson$1 or "step son$1" or "step-son$1" or famil* or "non famil*" or nonfamil* or "non-famil*" or "nonimmediate famil*" or "non immediate famil*" or "non-immediate famil*" or "non relative$1" or "non-relative" or nonrelative or unrelated or "un-related" or "non traditional" or nontraditional or "non-traditional" or "adopted child*" or "foster youth$1" or "foster child*" or friend$1 or neighbor$1 or companion$1 or acquaintance$1 or grandparent$1 or grandmother$1 or grandfather$1 or grandchild* or niece$1 or nephew$1 or sibling$1 or sister$1 or brother$1 or "sister in law" or "sister-in-law" or "sisters in law" or "sisters-in-law" or "brother in law" or "brother-in-law" or "brothers in law" or "brothers-in-law" or aunt$1 or uncle$1 or cousin$1 or exwife or "ex wife" or "ex-wife" or exwives or "ex wives" or "ex-wives" or "exhusband$1" or "ex

husband$1" or "ex-husband$1" or relative$1 or nonspousal or "non-spousal" or "non spousal" or nonmarital or "non marital" or "non-marital" or "extended famil*" or "family-of-choice" or "family of choice" or "families-of-choice" or "families of choice" or missionar* or "untrained personnel" or unpaid or informal or "community member$1" or "legal guardian$1" or "social support" or "social network$1" or "social resource$1" or "emotional support" or "non-spouse" or "non spouse" or nonspouse) adj5 (carer$1 or caregiver$1 or "care-giver" or "care giver$1" or caregiving or "care-partner" or "care partner$1" or "caregiving network$1" or volunteer* or "voluntary worker$1" or "household work" or housekeeping or housework or "household activit*")).ab,ti,kf.

6 4 or 5

7 Patient Reported Outcome Measures/ or exp Quality of life/ or Mental Health/ or exp Morale/ or Resilience, Psychological/ or Caregiver Burden/ or Compassion Fatigue/ or Self-Compassion/

8 ((patient report* or patient-report* or life quality or "health related quality of life" or HRQOL or "quality of life" or observer report* or observer-report* or clinician report* or clinician-report* or nurse report* or nurse-report* or self report* or self-report* or "well being" or wellbeing or well-being or burden or strain or anxiety or depression or stress or anger or "mental health" or morale or resilience or compassion fatigue or vicarious trauma* or secondary trauma* or self-compassion or self-forgiveness) adj4 (outcome$1 or questionnaire$1 or survey$1 or measure$1 or assessment$1 or instrument$1)).ab,ti,kf.

9 7 or 8

10 3 and 6 and 9

PsycInfo (EBSCOhost)

S1. DE "Dementia" OR DE "Vascular Dementia" OR DE "Dementia with Lewy Bodies" OR DE "AIDS Dementia Complex" OR DE "Alzheimer's Disease" OR DE "Amyotrophic Lateral Sclerosis" OR DE "Rett Syndrome" OR DE "Prion Diseases" OR DE "Frontotemporal Lobar Degeneration"

S2. TI("Pick Disease of the brain" OR "Pick's disease" OR "dementia with Lewy Bodies" OR "posterior cortical atrophy" OR amentia* OR dementia* OR alzheimer* OR aphasia? OR "mesulam syndrome" OR "mesulam's syndrome" OR "Creutzfeldt-Jakob Syndrome" OR "creutzfeldt ja?ob disease?" OR "creutzfeldt-ja?ob syndrome" OR "subacute spongiform encephalopath*" OR "Diffuse neurofibrillary tangles with calcification" OR "kosaka shibayam disease" OR "frontotemporal lobar degeneration?" OR ftld OR ftlds OR "Huntington? disease" OR "akinetic rigid variant of huntington disease" OR "chronic progressive hereditary chorea" OR "huntington? chorea" OR "huntington chronic progressive hereditary chorea" OR "chronic hereditary progressive chorea" OR "hereditary chronic progressive chorea" OR "Kluver-Bucy syndrome" OR "kluver bucy syndrome" OR "temporal lobectomy behavior syndrome" OR (binswanger* N5 (disease OR encephalopath*)) OR "lewy body disease" OR cadasil* OR "cerebral arteriopathy with subcortical infarcts and leukoencephalopathy" OR "cerebral autosomal dominant arteriopathy with subcortical infarcts and leukoencephalopathy" OR ALS OR "amyotrophic lateral sclerosis" OR "charcot disease" OR (gehrig* N1 disease) OR "guam disease" OR ((rett OR retts OR rett's) N1 (disorder* OR syndrome*)) OR (prion* N3 (disease*

OR disorder*)) OR "transmissible spongiform encephalopath*" OR "acquired immunodeficiency syndrome encephalopath*" OR "human immunodeficiency virus encephalopath*" OR ((AIDS OR HIV OR subcortical OR arteriosclerotic OR chronic OR progressive OR leuko) N4 encephalopath*) OR ((HIV OR AIDS) N4 "motor complex") OR ((ALS OR "amyotrophic lateral sclerosis") N15 Guam)) OR AB("Pick Disease of the brain" OR Pick's disease OR "dementia with Lewy Bodies" OR "posterior cortical atrophy or amentia*" OR dementia* OR alzheimer* OR aphasia? OR "mesulam syndrome" OR "mesulam's syndrome" OR "Creutzfeldt-Jakob Syndrome" OR "creutzfeldt ja?ob disease?" OR "creutzfeldt-ja?ob syndrome" OR "subacute spongiform encephalopath*" OR "Diffuse neurofibrillary tangles with calcification" OR "kosaka shibayam disease" OR "frontotemporal lobar degeneration?" OR ftld OR ftlds OR Huntington? disease OR "akinetic rigid variant of huntington disease" OR "chronic progressive hereditary chorea" OR "huntington? chorea" OR "huntington chronic progressive hereditary chorea" OR "chronic hereditary progressive chorea" OR "hereditary chronic progressive chorea" OR "Kluver-Bucy syndrome" OR "kluver bucy syndrome" or "temporal lobectomy behavior syndrome" OR (binswanger* N5 (disease or encephalopath*)) OR "lewy body disease" OR cadasil* OR "cerebral arteriopathy with subcortical infarcts and leukoencephalopathy" OR "cerebral autosomal dominant arteriopathy with subcortical infarcts and leukoencephalopathy" OR ALS OR "amyotrophic lateral sclerosis" OR "charcot disease" OR (gehrig* N1 disease) OR "guam disease" OR ((rett OR retts OR rett's) N1 (disorder* OR syndrome*)) OR (prion* N3 (disease* OR disorder*)) OR "transmissible spongiform encephalopath*" OR "acquired immunodeficiency syndrome encephalopath*" OR "human immunodeficiency virus encephalopath*" OR ((AIDS OR HIV OR subcortical OR arteriosclerotic OR chronic OR progressive OR leuko) N4 encephalopath*) OR ((HIV OR AIDS) N4 "motor complex") OR ((ALS OR "amyotrophic lateral sclerosis") N15 Guam))

S3. S1 OR S2

S4. DE "Family Structure" OR DE "Extended Family" OR DE "Stepfamily" OR DE "Stepchildren" OR DE "Stepparents" OR DE "Caregivers" OR DE "Adopted Children" OR DE "Foster Children" OR DE "Social Support" OR DE "Social Networks" OR DE "Social Resources" OR DE "Emotional Support" OR DE "Grandparents"

S5. TI(("family structure*" OR "reconstituted famil*" OR stepfamil* OR "step famil*" OR "step-famil*" OR "blended famil*" OR "step kin" OR "step-kin" OR "stepkin" OR stepparent? OR "step parent?" OR "step-parent?" OR stepdaughter? OR "step daughter?" OR "step-daughter?" OR stepson? OR "step son?" OR "step-son?" OR famil* OR "non famil*" OR nonfamil* OR "non-famil*" OR "nonimmediate famil*" OR "non immediate famil*" OR "non-immediate famil*" OR "non-relative" OR "non relative" OR "nonrelative" OR unrelated OR "un-related" OR nontraditional OR "non traditional" OR "non-traditional" OR "adopted child*" OR "foster youth?" OR "foster child*" OR friend? OR neighbor? OR companion? OR acquaintance? OR grandparent? OR grandmother? OR grandfather? OR grandchild* OR niece? OR nephew? OR sibling? OR sister? OR brother? OR "sister in law" OR "sister-in-law" OR "sisters in law" OR "sisters-in-law" OR "brother-in-law" OR "brother in law" OR "brothers-in-law" OR "brothers in law" OR aunt? OR uncle? OR cousin? OR "exwife" OR "ex wife" OR "ex-wife" OR "exwives" OR

"ex wives" OR "ex-wives" OR "exhusband?" OR "ex husband?" OR "ex-husband?" OR relative? OR "nonspousal" OR "non-spousal" OR "non spousal" OR "nonmarital" OR "non marital" OR "non-marital" OR "extended famil*" OR "family of choice" OR "family-of-choice" OR "families-of-choice" OR "families of choice" OR missionar* OR "untrained personnel" OR unpaid OR informal OR "community member?" OR "legal guardian?" OR "social support" OR "social network?" OR "social resource?" OR "emotional support" OR "non-spouse" OR "non spouse" OR "nonspouse") N5 (carer? OR caregiver? OR "care-giver" OR "care giver?" OR caregiving OR "care-partner?" OR "care partner?" OR "caregiving network?" OR volunteer* OR "voluntary worker?" OR "household work" OR "housekeeping" OR housework OR "household activit*")) OR AB(("family structure*" OR "reconstituted famil*" OR stepfamil* OR "step famil*" OR "step-famil*" OR "blended famil*" OR "step kin" OR "step-kin" OR "stepkin" OR stepparent? OR "step parent?" OR "step-parent?" OR stepdaughter? OR "step daughter?" OR "step-daughter?" OR stepson? OR "step son?" OR "step-son?" OR famil* OR "non famil*" OR nonfamil* OR "non-famil*" OR "nonimmediate famil*" OR "non immediate famil*" OR "non-immediate famil*" OR "non-relative" OR "non relative" OR "nonrelative" OR unrelated OR "un-related" OR nontraditional OR "non traditional" OR "non-traditional" OR "adopted child*" OR "foster youth?" OR "foster child*" OR friend? OR neighbor? OR companion? OR acquaintance? OR grandparent? OR grandmother? OR grandfather? OR grandchild* OR niece? OR nephew? OR sibling? OR sister? OR brother? OR "sister in law" OR "sister-in-law" OR "sisters in law" OR "sisters-in-law" OR "brother-in-law" OR "brother in law" OR "brothers-in-law" OR "brothers in law" OR aunt? OR uncle? OR cousin? OR "exwife" OR "ex wife" OR "ex-wife" OR "exwives" OR "ex wives" OR "ex-wives" OR "exhusband?" OR "ex husband?" OR "ex-husband?" OR relative? OR "nonspousal" OR "non-spousal" OR "non spousal" OR "nonmarital" OR "non marital" OR "non-marital" OR "extended famil*" OR "family of choice" OR "family-of-choice" OR "families-of-choice" OR "families of choice" OR missionar* OR "untrained personnel" OR unpaid OR informal OR "community member?" OR "legal guardian?" OR "social support" OR "social network?" OR "social resource?" OR "emotional support" OR "non-spouse" OR "non spouse" OR "nonspouse") N5 (carer? OR caregiver? OR "care-giver" OR "care giver?" OR caregiving OR "care-partner?" OR "care partner?" OR "caregiving network?" OR volunteer* OR "voluntary worker?" OR "household work" OR "housekeeping" OR housework OR "household activit*"))

S6. S4 OR S5

S7. DE "Patient Reported Outcome Measures" OR DE "Quality of Life" OR DE "Well Being" OR DE "Mental Health" OR DE "Morale" OR DE "Resilience (Psychological)" OR DE "Caregiver Burden" OR DE "Compassion Fatigue" OR DE "Self-Compassion" OR DE "Burnout" OR DE "Psychological Stress"

S8. TI(("patient reported" OR "patient-reported" OR "life quality" OR "health related quality of life" OR HRQOL OR "quality of life" OR "observer-report*" OR "observer report*" OR "clinician-report*" OR "clinician report*" OR "nurse-report*" OR "nurse report*" OR "self-report*" OR "self report*" OR "well-being" OR "well being" OR wellbeing OR burden OR strain OR anxiety OR depression OR stress OR anger OR "mental health" OR morale OR resilience OR "compassion fatigue" OR "secondary traumatic stress" OR burnout OR fatigue OR

"self-compassion" OR "self compassion") N4 (outcome? OR questionnaire? OR survey? OR measure? OR assessment? OR instrument?)) OR AB(("patient reported" OR "patient-reported" OR "life quality" OR "health related quality of life" OR HRQOL OR "quality of life" OR "observer-report*" OR "observer report*" OR "clinician-report*" OR "clinician report*" OR "nurse-report*" OR "nurse report*" OR "self-report*" OR "self report*" OR "well-being" OR "well being" OR wellbeing OR burden OR strain OR anxiety OR depression OR stress OR anger OR "mental health" OR morale OR resilience OR "compassion fatigue" OR "secondary traumatic stress" OR burnout OR fatigue OR "self-compassion" OR "self compassion") N4 (outcome? OR questionnaire? OR survey? OR measure? OR assessment? OR instrument?))

S9. S7 OR S8
S10. S3 AND S6 AND S9

AgeLine (EBSCOhost)

S1. DE "Dementia" OR DE "Alzheimers Disease" OR DE "Frontotemporal Dementia" OR DE "Lewy Body Dementia" OR DE "Vascular Dementia"

S2. TI("Pick Disease of the brain" OR Pick's disease OR "dementia with Lewy Bodies" OR posterior cortical atrophy OR amentia* OR dementia* OR alzheimer* OR aphasia? OR mesulam syndrome OR mesulam's syndrome OR Creutzfeldt-Jakob Syndrome OR creutzfeldt ja?ob disease? OR creutzfeldt-ja?ob syndrome OR subacute spongiform encephalopath* OR "Diffuse neurofibrillary tangles with calcification" OR kosaka shibayam disease OR frontotemporal lobar degeneration? OR ftld OR ftlds OR Huntington? disease OR "akinetic rigid variant of huntington disease" OR chronic progressive hereditary chorea OR huntington? chorea OR huntington chronic progressive hereditary chorea OR chronic hereditary progressive chorea OR hereditary chronic progressive chorea OR Kluver-Bucy syndrome OR kluver bucy syndrome OR temporal lobectomy behavior syndrome OR (binswanger* N5 (disease OR encephalopath*)) OR lewy body disease OR cadasil* OR "cerebral arteriopathy with subcortical infarcts and leukoencephalopathy" OR "cerebral autosomal dominant arteriopathy with subcortical infarcts and leukoencephalopathy" OR ALS OR amyotrophic lateral sclerosis OR charcot disease OR (gehrig* N1 disease) OR guam disease OR ((rett OR retts OR rett's) N1 (disorder* OR syndrome*)) OR (prion* N3 (disease* OR disorder*)) OR transmissible spongiform encephalopath* OR acquired immunodeficiency syndrome encephalopath* OR human immunodeficiency virus encephalopath* OR ((AIDS OR HIV OR subcortical OR arteriosclerotic OR chronic OR progressive OR leuko) N4 encephalopath*) OR ((HIV OR AIDS) N4 motor complex) OR ((ALS OR amyotrophic lateral sclerosis) N15 Guam)) OR AB("Pick Disease of the brain" OR Pick's disease OR "dementia with Lewy Bodies" OR posterior cortical atrophy or amentia* OR dementia* OR alzheimer* OR aphasia? OR mesulam syndrome OR mesulam's syndrome OR Creutzfeldt-Jakob Syndrome OR creutzfeldt ja?ob disease? OR creutzfeldt-ja?ob syndrome OR subacute spongiform encephalopath* OR "Diffuse neurofibrillary tangles with calcification" OR kosaka shibayam disease OR frontotemporal lobar degeneration? OR ftld OR ftlds OR Huntington? disease OR "akinetic rigid variant of huntington disease" OR chronic

progressive hereditary chorea OR huntington? chorea OR huntington chronic progressive hereditary chorea OR chronic hereditary progressive chorea OR hereditary chronic progressive chorea OR Kluver-Bucy syndrome OR kluver bucy syndrome or temporal lobectomy behavior syndrome OR (binswanger* N5 (disease or encephalopath*)) OR lewy body disease OR cadasil* OR "cerebral arteriopathy with subcortical infarcts and leukoencephalopathy" OR "cerebral autosomal dominant arteriopathy with subcortical infarcts and leukoencephalopathy" OR ALS OR amyotrophic lateral sclerosis OR charcot disease OR (gehrig* N1 disease) OR guam disease OR ((rett OR retts OR rett's) N1 (disorder* OR syndrome*)) OR (prion* N3 (disease* OR disorder*)) OR transmissible spongiform encephalopath* OR acquired immunodeficiency syndrome encephalopath* OR human immunodeficiency virus encephalopath* OR ((AIDS OR HIV OR subcortical OR arteriosclerotic OR chronic OR progressive OR leuko) N4 encephalopath*) OR ((HIV OR AIDS) N4 motor complex) OR ((ALS OR amyotrophic lateral sclerosis) N15 Guam))

S3. S1 OR S2

S4. DE "Extended Family" OR DE "Grandchildren" OR DE "Grandparents" OR DE "In Laws" OR DE "Parents" OR DE "Siblings" OR DE "Step Relatives" OR DE "Caregivers" OR DE "Siblings" OR DE "Informal Support Systems" OR DE "Guardianship" OR DE "Social Networks" OR DE "Emotional Support"
S5. TI(("family structure*" OR "reconstituted famil*" OR stepfamil* OR "step famil*" OR "step-famil*" OR "blended famil*" OR "step kin" OR "step-kin" OR "stepkin" OR stepparent? OR "step parent?" OR "step-parent?" OR stepdaughter? OR "step daughter?" OR "step-daughter?" OR stepson? OR "step son?" OR "step-son?" OR famil* OR "non famil*" OR nonfamil* OR "non-famil*" OR "nonimmediate famil*" OR "non immediate famil*" OR "non-immediate famil*" OR "non-relative" OR "non relative" OR "nonrelative" OR unrelated OR "un-related" OR nontraditional OR "non traditional" OR "non-traditional" OR "adopted child*" OR "foster youth?" OR "foster child*" OR friend? OR neighbor? OR companion? OR acquaintance? OR grandparent? OR grandmother? OR grandfather? OR grandchild* OR niece? OR nephew? OR sibling? OR sister? OR brother? OR "sister in law" OR "sister-in-law" OR "sisters in law" OR "sisters-in-law" OR "brother-in-law" OR "brother in law" OR "brothers-in-law" OR "brothers in law" OR aunt? OR uncle? OR cousin? OR "exwife" OR "ex wife" OR "ex-wife" OR "exwives" OR "ex wives" OR "ex-wives" OR "exhusband?" OR "ex husband?" OR "ex-husband?" OR relative? OR "nonspousal" OR "non-spousal" OR "non spousal" OR "nonmarital" OR "non marital" OR "non-marital" OR "extended famil*" OR "family of choice" OR "family-of-choice" OR "families-of-choice" OR "families of choice" OR missionar* OR "untrained personnel" OR unpaid OR informal OR "community member?" OR "legal guardian?" OR "social support" OR "social network?" OR "social resource?" OR "emotional support" OR "non-spouse" OR "non spouse" OR "nonspouse") N5 (carer? OR caregiver? OR "care-giver" OR "care giver?" OR caregiving OR "care-partner?" OR "care partner?" OR "caregiving network?" OR volunteer* OR "voluntary worker?" OR "household work" OR "housekeeping" OR housework OR "household activit*")) OR AB(("family structure*" OR "reconstituted famil*" OR stepfamil* OR "step famil*" OR "step-famil*" OR "blended famil*" OR "step kin" OR "step-kin" OR "stepkin" OR stepparent? OR "step parent?" OR "step-parent?" OR stepdaughter? OR "step daughter?" OR "step-daughter?"

OR stepson? OR "step son?" OR "step-son?" OR famil* OR "non famil*" OR nonfamil* OR "non-famil*" OR "nonimmediate famil*" OR "non immediate famil*" OR "non-immediate famil*" OR "non-relative" OR "non relative" OR "nonrelative" OR unrelated OR "un-related" OR nontraditional OR "non traditional" OR "non-traditional" OR "adopted child*" OR "foster youth?" OR "foster child*" OR friend? OR neighbor? OR companion? OR acquaintance? OR grandparent? OR grandmother? OR grandfather? OR grandchild* OR niece? OR nephew? OR sibling? OR sister? OR brother? OR "sister in law" OR "sister-in-law" OR "sisters in law" OR "sisters-in-law" OR "brother-in-law" OR "brother in law" OR "brothers-in-law" OR "brothers in law" OR aunt? OR uncle? OR cousin? OR "exwife" OR "ex wife" OR "ex-wife" OR "exwives" OR "ex wives" OR "ex-wives" OR "exhusband?" OR "ex husband?" OR "ex-husband?" OR relative? OR "nonspousal" OR "non-spousal" OR "non spousal" OR "nonmarital" OR "non marital" OR "non-marital" OR "extended famil*" OR "family of choice" OR "family-of-choice" OR "families-of-choice" OR "families of choice" OR missionar* OR "untrained personnel" OR unpaid OR informal OR "community member?" OR "legal guardian?" OR "social support" OR "social network?" OR "social resource?" OR "emotional support" OR "non-spouse" OR "non spouse" OR "nonspouse") N5 (carer? OR caregiver? OR "care-giver" OR "care giver?" OR caregiving OR "care-partner?" OR "care partner?" OR "caregiving network?" OR volunteer* OR "voluntary worker?" OR "household work" OR "housekeeping" OR housework OR "household activit*"))

S6. S4 OR S5

S7. DE "Outcomes" OR DE "Self Report Measures" OR DE "Quality of Life" OR DE "Psychological Well Being" OR DE "Mental Health" OR DE "Morale" OR DE "Resilience" OR DE "Caregiving Burden" OR DE "Psychological Well Being"

S8. TI(("patient reported" OR "patient-reported" OR "life quality" OR "health related quality of life" OR HRQOL OR "quality of life" OR "observer-report*" OR "observer report*" OR "clinician-report*" OR "clinician report*" OR "nurse-report*" OR "nurse report*" OR "self-report*" OR "self report*" OR "well-being" OR "well being" OR wellbeing OR burden OR strain OR anxiety OR depression OR stress OR anger OR "mental health" OR morale OR resilience OR "compassion fatigue" OR "secondary traumatic stress" OR burnout OR fatigue OR "self-compassion" OR "self compassion") N4 (outcome? OR questionnaire? OR survey? OR measure? OR assessment? OR instrument?)) OR AB(("patient reported" OR "patient-reported" OR "life quality" OR "health related quality of life" OR HRQOL OR "quality of life" OR "observer-report*" OR "observer report*" OR "clinician-report*" OR "clinician report*" OR "nurse-report*" OR "nurse report*" OR "self-report*" OR "self report*" OR "well-being" OR "well being" OR wellbeing OR burden OR strain OR anxiety OR depression OR stress OR anger OR "mental health" OR morale OR resilience OR "compassion fatigue" OR "secondary traumatic stress" OR burnout OR fatigue OR "self-compassion" OR "self compassion") N4 (outcome? OR questionnaire? OR survey? OR measure? OR assessment? OR instrument?))

S9. S7 OR S8
S10. S3 AND S6 AND S9

CINAHL Complete (EBSCOhost)

S1. (MH "Dementia") OR (MH "AIDS Dementia Complex") OR (MH "Dementia, Vascular") OR (MH "Lewy Body Disease") OR (MH "Mixed Dementias") OR (MH "Alzheimer's Disease") OR (MH "CADASIL") OR (MH "Amyotrophic Lateral Sclerosis") OR (MH "Rett Syndrome") OR (MH "Prion Diseases") OR (MH "Frontotemporal Dementia") OR (MH "Dementia, Multi-Infarct")

S2. TI("Pick Disease of the brain" OR "Pick's disease" OR "dementia with Lewy Bodies" OR "posterior cortical atrophy" OR amentia* OR dementia* OR alzheimer* OR aphasia? OR "mesulam syndrome" OR "mesulam's syndrome" OR "Creutzfeldt-Jakob Syndrome" OR "creutzfeldt ja?ob disease?" OR "creutzfeldt-ja?ob syndrome" OR "subacute spongiform encephalopath*" OR "Diffuse neurofibrillary tangles with calcification" OR "kosaka shibayam disease" OR "frontotemporal lobar degeneration?" OR ftld OR ftlds OR "Huntington? disease" OR "akinetic rigid variant of huntington disease" OR "chronic progressive hereditary chorea" OR "huntington? chorea" OR "huntington chronic progressive hereditary chorea" OR "chronic hereditary progressive chorea" OR "hereditary chronic progressive chorea" OR "Kluver-Bucy syndrome" OR "kluver bucy syndrome" OR "temporal lobectomy behavior syndrome" OR (binswanger* N5 (disease OR encephalopath*)) OR "lewy body disease" OR cadasil* OR "cerebral arteriopathy with subcortical infarcts and leukoencephalopathy" OR "cerebral autosomal dominant arteriopathy with subcortical infarcts and leukoencephalopathy" OR ALS OR "amyotrophic lateral sclerosis" OR "charcot disease" OR (gehrig* N1 disease) OR "guam disease" OR ((rett OR retts OR rett's) N1 (disorder* OR syndrome*)) OR (prion* N3 (disease* OR disorder*)) OR "transmissible spongiform encephalopath*" OR "acquired immunodeficiency syndrome encephalopath*" OR "human immunodeficiency virus encephalopath*" OR ((AIDS OR HIV OR subcortical OR arteriosclerotic OR chronic OR progressive OR leuko) N4 encephalopath*) OR ((HIV OR AIDS) N4 "motor complex") OR ((ALS OR "amyotrophic lateral sclerosis") N15 Guam)) OR AB("Pick Disease of the brain" OR Pick's disease OR "dementia with Lewy Bodies" OR "posterior cortical atrophy or amentia*" OR dementia* OR alzheimer* OR aphasia? OR "mesulam syndrome" OR "mesulam's syndrome" OR "Creutzfeldt-Jakob Syndrome" OR "creutzfeldt ja?ob disease?" OR "creutzfeldt-ja?ob syndrome" OR "subacute spongiform encephalopath*" OR "Diffuse neurofibrillary tangles with calcification" OR "kosaka shibayam disease" OR "frontotemporal lobar degeneration?" OR ftld OR ftlds OR Huntington? disease OR "akinetic rigid variant of huntington disease" OR "chronic progressive hereditary chorea" OR "huntington? chorea" OR "huntington chronic progressive hereditary chorea" OR "chronic hereditary progressive chorea" OR "hereditary chronic progressive chorea" OR "Kluver-Bucy syndrome" OR "kluver bucy syndrome" or "temporal lobectomy behavior syndrome" OR (binswanger* N5 (disease or encephalopath*)) OR "lewy body disease" OR cadasil* OR "cerebral arteriopathy with subcortical infarcts and leukoencephalopathy" OR "cerebral autosomal dominant arteriopathy with subcortical infarcts and leukoencephalopathy" OR ALS OR "amyotrophic lateral sclerosis" OR "charcot disease" OR (gehrig* N1 disease) OR "guam disease" OR ((rett OR retts OR rett's) N1 (disorder* OR syndrome*)) OR (prion* N3 (disease* OR disorder*)) OR "transmissible spongiform encephalopath*" OR "acquired immunodeficiency syndrome encephalopath*" OR "human immunodeficiency virus encephalopath*" OR ((AIDS OR HIV OR subcortical OR arteriosclerotic OR chronic OR

progressive OR leuko) N4 encephalopath*) OR ((HIV OR AIDS) N4 "motor complex") OR ((ALS OR "amyotrophic lateral sclerosis") N15 Guam))

S3. S1 OR S2

S4. (MH "Extended Family") OR (MH "Stepfamilies") OR (MH "Caregivers") OR (MH "Siblings") OR (MH "Child, Adopted") OR (MH "Child, Foster") OR (MH "Grandparents") OR (MH "Guardianship, Legal") OR (MH "Social Support") OR (MH "Social Networks")

S5. TI(("family structure*" OR "reconstituted famil*" OR stepfamil* OR "step famil*" OR "step-famil*" OR "blended famil*" OR "step kin" OR "step-kin" OR "stepkin" OR stepparent? OR "step parent?" OR "step-parent?" OR stepdaughter? OR "step daughter?" OR "step-daughter?" OR stepson? OR "step son?" OR "step-son?" OR famil* OR "non famil*" OR nonfamil* OR "non-famil*" OR "nonimmediate famil*" OR "non immediate famil*" OR "non-immediate famil*" OR "non-relative" OR "non relative" OR "nonrelative" OR unrelated OR "un-related" OR nontraditional OR "non traditional" OR "non-traditional" OR "adopted child*" OR "foster youth?" OR "foster child*" OR friend? OR neighbor? OR companion? OR acquaintance? OR grandparent? OR grandmother? OR grandfather? OR grandchild* OR niece? OR nephew? OR sibling? OR sister? OR brother? OR "sister in law" OR "sister-in-law" OR "sisters in law" OR "sisters-in-law" OR "brother-in-law" OR "brother in law" OR "brothers-in-law" OR "brothers in law" OR aunt? OR uncle? OR cousin? OR "exwife" OR "ex wife" OR "ex-wife" OR "exwives" OR "ex wives" OR "ex-wives" OR "exhusband?" OR "ex husband?" OR "ex-husband?" OR relative? OR "nonspousal" OR "non-spousal" OR "non spousal" OR "nonmarital" OR "non marital" OR "non-marital" OR "extended famil*" OR "family of choice" OR "family-of-choice" OR "families-of-choice" OR "families of choice" OR missionar* OR "untrained personnel" OR unpaid OR informal OR "community member?" OR "legal guardian?" OR "social support" OR "social network?" OR "social resource?" OR "emotional support" OR "non-spouse" OR "non spouse" OR "nonspouse") N5 (carer? OR caregiver? OR "care-giver" OR "care giver?" OR caregiving OR "care-partner?" OR "care partner?" OR "caregiving network?" OR volunteer* OR "voluntary worker?" OR "household work" OR "housekeeping" OR housework OR "household activit*")) OR AB(("family structure*" OR "reconstituted famil*" OR stepfamil* OR "step famil*" OR "step-famil*" OR "blended famil*" OR "step kin" OR "step-kin" OR "stepkin" OR stepparent? OR "step parent?" OR "step-parent?" OR stepdaughter? OR "step daughter?" OR "step-daughter?" OR stepson? OR "step son?" OR "step-son?" OR famil* OR "non famil*" OR nonfamil* OR "non-famil*" OR "nonimmediate famil*" OR "non immediate famil*" OR "non-immediate famil*" OR "non-relative" OR "non relative" OR "nonrelative" OR unrelated OR "un-related" OR nontraditional OR "non traditional" OR "non-traditional" OR "adopted child*" OR "foster youth?" OR "foster child*" OR friend? OR neighbor? OR companion? OR acquaintance? OR grandparent? OR grandmother? OR grandfather? OR grandchild* OR niece? OR nephew? OR sibling? OR sister? OR brother? OR "sister in law" OR "sister-in-law" OR "sisters in law" OR "sisters-in-law" OR "brother-in-law" OR "brother in law" OR "brothers-in-law" OR "brothers in law" OR aunt? OR uncle? OR cousin? OR "exwife" OR "ex wife" OR "ex-wife" OR "exwives" OR "ex wives" OR "ex-wives" OR "exhusband?" OR "ex husband?" OR "ex-husband?" OR relative? OR "nonspousal" OR "non-spousal" OR "non spousal" OR "nonmarital" OR "non marital" OR

"non-marital" OR "extended famil*" OR "family of choice" OR "family-of-choice" OR "families-of-choice" OR "families of choice" OR missionar* OR "untrained personnel" OR unpaid OR informal OR "community member?" OR "legal guardian?" OR "social support" OR "social network?" OR "social resource?" OR "emotional support" OR "non-spouse" OR "non spouse" OR "nonspouse") N5 (carer? OR caregiver? OR "care-giver" OR "care giver?" OR caregiving OR "care-partner?" OR "care partner?" OR "caregiving network?" OR volunteer* OR "voluntary worker?" OR "household work" OR "housekeeping" OR housework OR "household activit*"))

S6. S4 OR S5

S7. (MH "Patient-Reported Outcomes") OR (MH "Quality of Life") OR (MH "Psychological Well-Being") OR (MH "Mental Health") OR (MH "Morale") OR (MH "Hardiness") OR (MH "Caregiver Burden") OR (MH "Compassion Fatigue") OR (MH "Self-Compassion") OR (MH "Stress Psychological")

S8. TI(("patient reported" OR "patient-reported" OR "life quality" OR "health related quality of life" OR HRQOL OR "quality of life" OR "observer-report*" OR "observer report*" OR "clinician-report*" OR "clinician report*" OR "nurse-report*" OR "nurse report*" OR "self-report*" OR "self report*" OR "well-being" OR "well being" OR wellbeing OR burden OR strain OR anxiety OR depression OR stress OR anger OR "mental health" OR morale OR resilience OR "compassion fatigue" OR "secondary traumatic stress" OR burnout OR fatigue OR "self-compassion" OR "self compassion") N4 (outcome? OR questionnaire? OR survey? OR measure? OR assessment? OR instrument?)) OR AB(("patient reported" OR "patient-reported" OR "life quality" OR "health related quality of life" OR HRQOL OR "quality of life" OR "observer-report*" OR "observer report*" OR "clinician-report*" OR "clinician report*" OR "nurse-report*" OR "nurse report*" OR "self-report*" OR "self report*" OR "well-being" OR "well being" OR wellbeing OR burden OR strain OR anxiety OR depression OR stress OR anger OR "mental health" OR morale OR resilience OR "compassion fatigue" OR "secondary traumatic stress" OR burnout OR fatigue OR "self-compassion" OR "self compassion") N4 (outcome? OR questionnaire? OR survey? OR measure? OR assessment? OR instrument?))

S9. S7 OR S8
S10. S3 AND S6 AND S9
