# Supplementary material for: Understanding how informal dementia caregiver networks are assessed in the literature: results from a scoping review
Source: Front Dement. 2026 Jun 12;5:1824829. doi: 10.3389/frdem.2026.1824829 (PMC13303008; doi:10.3389/frdem.2026.1824829)
Supplement: Supplementary file 2 [file Table_1.DOCX]

**References for Articles that Included a Caregiver Network Measure or Assessment**

Abdollahpour, I., Nedjat, S., Salimi, Y., Noroozian, M., & Majdzadeh, R. (2015). Which variable is the strongest adjusted predictor of quality of life in caregivers of patients with dementia? Psychogeriatrics : the official journal of the Japanese Psychogeriatric Society, 15(1), 51-57. https://doi.org/https://dx.doi.org/10.1111/psyg.12094

Ali, T., McAvay, G. J., Monin, J. K., & Gill, T. M. (2022). Patterns of Caregiving Among Older Adults With and Without Dementia: A Latent Class Analysis. Journals of Gerontology Series B: Psychological Sciences & Social Sciences, 77, S74-S85. https://doi.org/10.1093/geronb/gbab237

Altieri, M., & Santangelo, G. (2021). The Psychological Impact of COVID-19 Pandemic and Lockdown on Caregivers of People With Dementia. The American journal of geriatric psychiatry : official journal of the American Association for Geriatric Psychiatry, 29(1), 27-34. https://doi.org/https://dx.doi.org/10.1016/j.jagp.2020.10.009

Aminzadeh, F., Byszewski, A., & Dalziel, W. B. (2006). A prospective study of caregiver burden in an outpatient comprehensive geriatric assessment program. Clinical Gerontologist, 29(4), 47-60. https://doi.org/10.1300/j018v29n04_04

Arai, Y., Zarit, S. H., Sugiura, M., & Washio, M. (2002). Patterns of outcome of caregiving for the impaired elderly: A longitudinal study in rural Japan. Aging & Mental Health, 6(1), 39-46. https://doi.org/10.1080/13607860120101059

Ashida, S., Marcum, C. S., & Koehly, L. M. (2018). Unmet Expectations in Alzheimer's Family Caregiving: Interactional Characteristics Associated With Perceived Under-Contribution. Gerontologist, 58(2), e46-e55. https://doi.org/10.1093/geront/gnx141

Au, A., Lau, K. M., Koo, S., Cheung, G., Pan, P. C., & Wong, M. K. (2009). The effects of informal social support on depressive symptoms and life satisfaction in dementia caregivers in Hong Kong. Hong Kong Journal of Psychiatry, 19(2), 57-64. http://proxy.lib.umich.edu/login?url=https://search.ebscohost.com/login.aspx?direct=true&db=psyh&AN=2009-09162-002&site=ehost-live&scope=site

Baker, K. L., Robertson, N., & Connelly, D. (2010). Men caring for wives or partners with dementia: Masculinity, strain and gain. Aging & Mental Health, 14(3), 319-327. https://doi.org/10.1080/13607860903228788

Bass, D. M., McClendon, M. J., Brennan, P. F., & McCarthy, C. (1998). The buffering effect of a computer support network on caregiver strain. Journal of aging and health, 10(1), 20-43. https://doi.org/10.1177/089826439801000102

Bédard, M., Kuzik, R., Chambers, L., Molloy, D. W., Dubois, S., & Lever, J. A. (2005). Understanding burden differences between men and women caregivers: The contribution of care-recipient problem behaviors. International Psychogeriatrics, 17(1), 99-118. https://doi.org/10.1017/S1041610204000857

Bedard, M., Molloy, D. W., Pedlar, D., Lever, J. A., & Stones, M. J. (1997). Associations between dysfunctional behaviors, gender, and burden in spousal caregivers of cognitively impaired older adults. International Psychogeriatrics, 9(3), 277-290. http://proxy.lib.umich.edu/login?url=https://search.ebscohost.com/login.aspx?direct=true&db=gnh&AN=73676&site=ehost-live&scope=site

Bejjani, C., Snow, A. L., Judge, K. S., Bass, D. M., Morgan, R. O., Wilson, N., Walder, A., Looman, W. J., McCarthy, C., & Kunik, M. E. (2015). Characteristics of Depressed Caregivers of Veterans With Dementia. American journal of Alzheimer's disease and other dementias, 30(7), 672-678. https://doi.org/https://dx.doi.org/10.1177/1533317512461555

Bekhet, A. K. (2013). Effects of positive cognitions and resourcefulness on caregiver burden among caregivers of persons with dementia. International journal of mental health nursing, 22(4), 340-346. https://doi.org/10.1111/j.1447-0349.2012.00877.x

Bell, J. F., Whitney, R. L., Keeton, V., & Young, H. M. (2021). Caregiver Characteristics and Outcomes Associated With Level of Care Complexity for Older Adults. Research in Gerontological Nursing, 14(3), 117-125. https://doi.org/10.3928/19404921-20210427-01

Bergman-Evans, B. (1994). A health profile of spousal Alzheimer's caregivers: depression and physical health characteristics. Journal of Psychosocial Nursing & Mental Health Services, 32(9), 25-49. http://proxy.lib.umich.edu/login?url=https://search.ebscohost.com/login.aspx?direct=true&db=ccm&AN=107455774&site=ehost-live&scope=site

Biegel, D. E., Bass, D. M., Schulz, R., & Morycz, R. (1993). Predictors of in-home and out-of-home service use by family caregivers of Alzheimer's disease patients. Journal of Aging & Health, 5(4), 419-438. https://doi.org/10.1177/089826439300500401

Bindoff, H. P., Clifford, C. A., & Young, J. L. (1997). Caregivers of family members with dementia and disability: A comparative study of wellbeing. Journal of Family Studies, 3(2), 183-195. https://doi.org/10.5172/jfs.3.2.183

Birkel, R. C. (1987). Toward a social ecology of the home-care household. Psychology and aging, 2(3), 294-301. https://doi.org/10.1037/0882-7974.2.3.294

Boogaard, J. A., van der Steen, J. T., de Boer, A. H., & van Groenou, M. I. B. (2019). How Is End-of-Life Care With and Without Dementia Associated With Informal Caregivers' Outcomes? The American journal of hospice & palliative care, 36(11), 1008-1015. https://doi.org/https://dx.doi.org/10.1177/1049909119836932

Bruvik, F. K., Ulstein, I. D., Ranhoff, A. H., & Engedal, K. (2012). The quality of life of people with dementia and their family carers. Dementia and geriatric cognitive disorders, 34(1), 7-14. https://doi.org/https://dx.doi.org/10.1159/000341584

Buckwalter, K. C., Gerdner, L., Kohout, F., Hall, G. R., Kelly, A., Richards, B., & Sime, M. (1999). A nursing intervention to decrease depression in family caregivers of persons with dementia. Archives of psychiatric nursing, 13(2), 80-88. http://ovidsp.ovid.com/ovidweb.cgi?T=JS&PAGE=reference&D=med4&NEWS=N&AN=10222636

Buhr, G. T., Kuchibhatla, M., & Clipp, E. C. (2006). Caregivers' Reasons for Nursing Home Placement: Clues for Improving Discussions With Families Prior to the Transition. The Gerontologist, 46(1), 52-61. https://doi.org/10.1093/geront/46.1.52

Burgener, S. C., Bakas, T., Murray, C., Dunahee, J., & Tossey, S. (1998). Effective caregiving approaches for patients with Alzheimer's disease. Geriatric Nursing, 19(3), 121-126. http://proxy.lib.umich.edu/login?url=https://search.ebscohost.com/login.aspx?direct=true&db=gnh&AN=73491&site=ehost-live&scope=site

Canadian Study of Health and Aging Workgroup. (2002). Patterns and health effects of caring for people with dementia: The impact of changing cognitive and residential status. The Gerontologist, 42(5), 643-652. https://doi.org/10.1093/geront/42.5.643

Caserta, M. S., Lund, D. A., Wright, S. D., & Redburn, D. E. (1987). Caregivers to dementia patients: the utilization of community services. Gerontologist, 27(2), 209-214. http://proxy.lib.umich.edu/login?url=https://search.ebscohost.com/login.aspx?direct=true&db=gnh&AN=41265&site=ehost-live&scope=site

Cavallo, M. C., & Fattore, G. (1997). Economic and social burden of Alzheimer disease on families in the Lombardy region of Italy. Alzheimer disease and associated disorders, 11(4), 184-190. http://proxy.lib.umich.edu/login?url=https://search.ebscohost.com/login.aspx?direct=true&db=gnh&AN=72147&site=ehost-live&scope=site

Chappell, N. L., Dujela, C., & Smith, A. (2015). Caregiver Well-Being: Intersections of Relationship and Gender. Research on aging, 37(6), 623-645. https://doi.org/https://dx.doi.org/10.1177/0164027514549258

Chiu, L., Shyu, W., Liu, Y., Wang, S., & Chang, T. (2001). Factors determining the attitudes of family caregivers of dementia patients toward nursing home placement in Taiwan: comparisons between urban and semiurban areas. Public Health Nursing, 18(4), 281-291. https://doi.org/10.1046/j.1525-1446.2001.00281.x

Cho, J., Ory, M. G., & Stevens, A. B. (2016). Socioecological factors and positive aspects of caregiving: Findings from the REACH II intervention. Aging & Mental Health, 20(11), 1190-1201. https://doi.org/10.1080/13607863.2015.1068739

Chun, M., Knight, B. G., & Youn, G. (2007). Differences in stress and coping models of emotional distress among Korean, Korean-American and White-American caregivers. Aging & Mental Health, 11(1), 20-29. https://doi.org/10.1080/13607860600736232

Cody, P., Montgomery, A. J., Gray, F. C., Saunders-Goldson, S., & Baker, S. R. (2021). Caregiver Burdens of Family Members with Alzheimer's Disease. Journal of National Black Nurses' Association : JNBNA, 32(1), 41-48. http://ovidsp.ovid.com/ovidweb.cgi?T=JS&PAGE=reference&D=med20&NEWS=N&AN=34562352

Cohen, C. A., Gold, D. P., Shulman, K. I., Wortley, J. T., & McDonald, G. (1993). Factors determining the decision to institutionalize dementing individuals: a prospective study. Gerontologist, 33(6), 714-720. http://proxy.lib.umich.edu/login?url=https://search.ebscohost.com/login.aspx?direct=true&db=gnh&AN=59361&site=ehost-live&scope=site

Colerick, E. J., & George, L. K. (1986). Predictors of institutionalization among caregivers of patients with Alzheimer's disease. Journal of the American Geriatrics Society, 34(7), 493-498. https://doi.org/10.1111/j.1532-5415.1986.tb04239.x

Contreras, M., Mioshi, E., & Kishita, N. (2021). Factors predicting quality of life in family carers of people with dementia: The role of psychological inflexibility. Journal of Contextual Behavioral Science, 22, 7-12. https://doi.org/10.1016/j.jcbs.2021.08.003

Covinsky, K. E., Eng, C., Lui, L.-Y., Sands, L. P., Sehgal, A. R., Walter, L. C., Wieland, D., Eleazer, G. P., & Yaffe, K. (2001). Reduced employment in caregivers of frail elders: impact of ethnicity, patient clinical characteristics, and caregiver characteristics. Journals of Gerontology: Series A: Biological Sciences and Medical Sciences, 56A(11), M707-M713. http://proxy.lib.umich.edu/login?url=https://search.ebscohost.com/login.aspx?direct=true&db=gnh&AN=87841&site=ehost-live&scope=site

Cox, C. (1995). Comparing the experiences of Black and White caregivers of dementia patients. Social Work, 40(3), 343-349. http://proxy.lib.umich.edu/login?url=https://search.ebscohost.com/login.aspx?direct=true&db=psyh&AN=1995-41663-001&site=ehost-live&scope=site

Cox, C. (1998). Experience of respite: meeting the needs of African American and white caregivers in a statewide program. Journal of Gerontological Social Work, 30(3-4), 59-72. http://proxy.lib.umich.edu/login?url=https://search.ebscohost.com/login.aspx?direct=true&db=gnh&AN=76815&site=ehost-live&scope=site

Cox, C. (1999). Race and caregiving: patterns of service use by African American and white caregivers of persons with Alzheimer's disease. Journal of Gerontological Social Work, 32(2), 5-19. http://proxy.lib.umich.edu/login?url=https://search.ebscohost.com/login.aspx?direct=true&db=gnh&AN=79102&site=ehost-live&scope=site

Cox, C., & Monk, A. (1990). Minority caregivers of dementia victims: A comparison of Black and Hispanic families. Journal of Applied Gerontology, 9(3), 340-354. https://doi.org/10.1177/073346489000900308

Cox, C., & Monk, A. (1996). Strain among caregivers: Comparing the experiences of African American and Hispanic caregivers of Alzheimer's relatives. The International Journal of Aging & Human Development, 43(2), 93-106. https://doi.org/10.2190/DYQ1-TPRP-VHTC-38VU

Cox, C., & Verdieck, M. J. (1994). Factors affecting the outcomes of hospitalized dementia patients: from home to hospital to discharge. Gerontologist, 34(4), 497-504. http://proxy.lib.umich.edu/login?url=https://search.ebscohost.com/login.aspx?direct=true&db=gnh&AN=61301&site=ehost-live&scope=site

Custodio, N., Lira, D., Herrera-Perez, E., del Prado, L. N., Parodi, J., Guevara-Silva, E., Castro-Suarez, S., Mar, M., Montesinos, R., & Cortijo, P. (2014). Informal caregiver burden in middle-income countries: Results from Memory Centers in Lima—Peru. Dementia & neuropsychologia, 8(4), 376-383. https://doi.org/10.1590/S1980-57642014DN84000012

Damian, A. C., Mihailescu, A. I., Anghele, C., Ciobanu, C. A., Petrescu, C., Riga, S., Dionisie, V., & Ciobanu, A. M. (2023). Quality of Life Predictors in a Group of Informal Caregivers during the COVID-19 Pandemic. Medicina (Kaunas, Lithuania), 59(8). https://doi.org/https://dx.doi.org/10.3390/medicina59081486

Davis, L. L., Weaver, M., & Habermann, B. (2006). Differential attrition in a caregiver skill training trial. Research in Nursing & Health, 29(5), 498-506. https://doi.org/10.1002/nur.20138

de Labra, C., Millán-Calenti, J. C., Buján, A., Núñez-Naveira, L., Jensen, A. M., Peersen, M. C., Mojs, E., Samborski, W., & Maseda, A. (2015). Predictors of caregiving satisfaction in informal caregivers of people with dementia. Archives of gerontology and geriatrics, 60(3), 380-388. https://doi.org/10.1016/j.archger.2015.03.002

Dreyer, J., Bergmann, J. M., Kohler, K., Hochgraeber, I., Pinkert, C., Roes, M., Thyrian, J. R., Wiegelmann, H., & Holle, B. (2022). Differences and commonalities of home-based care arrangements for persons living with dementia in Germany - a theory-driven development of types using multiple correspondence analysis and hierarchical cluster analysis. BMC Geriatrics, 22(1), 723. https://doi.org/https://dx.doi.org/10.1186/s12877-022-03310-1

Elmståhl, S., Dahlrup, B., Ekström, H., & Nordell, E. (2018). The association between medical diagnosis and caregiver burden: a cross-sectional study of recipients of informal support and caregivers from the general population study ‘Good Aging in Skåne’, Sweden. Aging Clinical & Experimental Research, 30(9), 1023-1032. https://doi.org/10.1007/s40520-017-0870-0

Fang, B., Liu, H., & Yan, E. (2021). Association Between Caregiver Depression and Elder Mistreatment-Examining the Moderating Effect of Care Recipient Neuropsychiatric Symptoms and Caregiver-Perceived Burden. The journals of gerontology. Series B, Psychological sciences and social sciences, 76(10), 2098-2111. https://doi.org/https://dx.doi.org/10.1093/geronb/gbab025

Ferri, C. P., Ames, D., Prince, M., & Dementia Research, G. (2004). Behavioral and psychological symptoms of dementia in developing countries. Int Psychogeriatr, 16(4), 441-459. https://doi.org/10.1017/s1041610204000833

Ferris, S. H., Steinberg, G., Shulman, E., Kahn, R., & Reisberg, B. (1987). Institutionalization of Alzheimer's disease patients: reducing precipitating factors through family counseling. Home health care services quarterly, 8(1), 23-51. http://proxy.lib.umich.edu/login?url=https://search.ebscohost.com/login.aspx?direct=true&db=gnh&AN=42070&site=ehost-live&scope=site

Fialho, P. P. A., Koenig, A. M., Santos, M. D. L. d., Barbosa, M. T., & Caramelli, P. (2012). Positive effects of a cognitive-behavioral intervention program for family caregivers of demented elderly. Arquivos de neuro-psiquiatria, 70(10), 786-792. http://ovidsp.ovid.com/ovidweb.cgi?T=JS&PAGE=reference&D=med9&NEWS=N&AN=23060105

Gallagher-Thompson, D., Coon, D. W., Solano, N., Ambler, C., Rabinowitz, Y., & Thompson, L. W. (2003). Change in Indices of Distress Among Latino and Anglo Female Caregivers of Elderly Relatives With Dementia: Site-Specific Results From the REACH National Collaborative Study. The Gerontologist, 43(4), 580-591. https://doi.org/10.1093/geront/43.4.580

Gallicchio, L., Siddiqi, N., Langenberg, P., & Baumgarten, M. (2002). Gender differences in burden and depression among informal caregivers of demented elders in the community. International Journal of Geriatric Psychiatry, 17(2), 154-163. https://doi.org/10.1002/gps.538

Garand, L., Buckwalter, K. C., Lubaroff, D., Tripp-Reimer, T., Frantz, R. A., & Ansley, T. N. (2002). A pilot study of immune and mood outcomes of a community-based intervention for dementia caregivers: The PLST intervention. Archives of psychiatric nursing, 16(4), 156-167. https://doi.org/10.1053/apnu.2002.34392

Garcia, M. A., Diminich, E. D., Lu, P., Arevalo, S. P., Sayed, L., Abdelrahim, R., & Ajrouch, K. J. (2023). Caregiving for Foreign-Born Older Adults With Dementia. The journals of gerontology. Series B, Psychological sciences and social sciences, 78(Suppl 1), S4-S14. https://doi.org/https://dx.doi.org/10.1093/geronb/gbac153

Garre-Olmo, J., Vilalta-Franch, J., Calvo-Perxas, L., Turro-Garriga, O., Conde-Sala, L., & Lopez-Pousa, S. (2016). A path analysis of patient dependence and caregiver burden in Alzheimer's disease. International Psychogeriatrics, 28(7), 1133-1141. https://doi.org/https://dx.doi.org/10.1017/S1041610216000223

Garzon-Maldonado, F. J., Gutierrez-Bedmar, M., Garcia-Casares, N., Perez-Errazquin, F., Gallardo-Tur, A., & Martinez-Valle Torres, M. D. (2017). Health-related quality of life in caregivers of patients with Alzheimer's disease. Calidad de vida relacionada con la salud en cuidadores de pacientes con enfermedad de Alzheimer., 32(8), 508-515. https://doi.org/https://dx.doi.org/10.1016/j.nrl.2016.02.023

Gaugler, J. E., Anderson, K. A., Leach, C. R., Smith, C. D., Schmitt, F. A., & Mendiondo, M. (2004). The emotional ramifications of unmet need in dementia caregiving. American journal of Alzheimer's disease and other dementias, 19(6), 369-378. https://doi.org/10.1177/153331750401900605

Gaugler, J. E., Jarrott, S. E., Zarit, S. H., Stephens, M.-A. P., Townsend, A., & Greene, R. (2003). Adult day service use and reductions in caregiving hours: effects on stress and psychological well-being for dementia caregivers. International Journal of Geriatric Psychiatry, 18(1), 55-62. http://proxy.lib.umich.edu/login?url=https://search.ebscohost.com/login.aspx?direct=true&db=gnh&AN=92716&site=ehost-live&scope=site

Gaugler, J. E., Jarrott, S. E., Zarit, S. H., Stephens, M. P., Townsend, A., Greene, R., Gaugler, J. E., Jarrott, S. E., Zarit, S. H., Stephens, M.-A. P., Townsend, A., & Greene, R. (2003). Respite for dementia caregivers: the effects of adult day service use on caregiving hours and care demands. International Psychogeriatrics, 37-58. http://proxy.lib.umich.edu/login?url=https://search.ebscohost.com/login.aspx?direct=true&db=ccm&AN=106878675&site=ehost-live&scope=site

Gaugler, J. E., Kane, R. L., Kane, R. A., & Newcomer, R. (2005). Longitudinal effects of early behavior problems in the dementia caregiving career. Psychology and aging, 20(1), 100-116. http://proxy.lib.umich.edu/login?url=https://search.ebscohost.com/login.aspx?direct=true&db=gnh&AN=107126&site=ehost-live&scope=site

Gaugler, J. E., Kane, R. L., Kane, R. A., & Newcomer, R. (2005). Unmet care needs and key outcomes in dementia. Journal of the American Geriatrics Society, 53(12), 2098-2105. https://doi.org/10.1111/j.1532-5415.2005.00495.x

Gaugler, J. E., Kane, R. L., & Newcomer, R. (2007). Resilience and transitions from dementia caregiving. The Journals of Gerontology: Series B: Psychological Sciences and Social Sciences, 62(1), P38-P44. https://doi.org/10.1093/geronb/62.1.P38

Gaugler, J. E., Mittelman, M. S., Hepburn, K., & Newcomer, R. (2009). Predictors of change in caregiver burden and depressive symptoms following nursing home admission. Psychology and aging, 24(2), 385-396. https://doi.org/https://dx.doi.org/10.1037/a0016052

Gaugler, J. E., Mittelman, M. S., Hepburn, K., & Newcomer, R. (2010). Clinically significant changes in burden and depression among dementia caregivers following nursing home admission. BMC medicine, 8, 85. https://doi.org/https://dx.doi.org/10.1186/1741-7015-8-85

Gaugler, J. E., Mittelman, M. S., Hepburn, K., & Newcomer, R. (2014). Identifying at-risk dementia caregivers following institutionalization: The nursing home admission-burden and nursing home admission-depression prognostic tools. Journal of Applied Gerontology, 33(5), 624-646. https://doi.org/10.1177/0733464812454008

Gitlin, L. N., Hauck, W. W., Dennis, M. P., & Winter, L. (2005). Maintenance of effects of the home environmental skill-building program for family caregivers and individuals with Alzheimer's disease and related disorders. The journals of gerontology. Series A, Biological sciences and medical sciences, 60(3), 368-374. http://ovidsp.ovid.com/ovidweb.cgi?T=JS&PAGE=reference&D=med6&NEWS=N&AN=15860476

Gitlin, L. N., Winter, L., Corcoran, M., Dennis, M. P., Schinfeld, S., & Hauck, W. W. (2003). Effects of the home environmental skill-building program on the caregiver-care recipient dyad: 6-month outcomes from the Philadelphia REACH Initiative. The Gerontologist, 43(4), 532-546. http://ovidsp.ovid.com/ovidweb.cgi?T=JS&PAGE=reference&D=med5&NEWS=N&AN=12937332

Goodman, C. C., & Pynoos, J. (1990). Model telephone information and support program for caregivers of Alzheimer's patients. Gerontologist, 30(3), 399-404. http://proxy.lib.umich.edu/login?url=https://search.ebscohost.com/login.aspx?direct=true&db=gnh&AN=49921&site=ehost-live&scope=site

Grafström, M., & Winblad, B. (1995). Family burden in the care of the demented and nondemented elderly: A longitudinal study. Alzheimer disease and associated disorders, 9(2), 78-86. https://doi.org/10.1097/00002093-199509020-00004

Hannappel, M., Calsyn, R. J., & Allen, G. (1993). Does social support alleviate the depression of caregivers of dementia patients? Journal of Gerontological Social Work, 20(1/2), 35-51. https://doi.org/10.1300/j083v20n01_04

Herrera, A. P., Lee, J. W., Nanyonjo, R. D., Laufman, L. E., & Torres-Vigil, I. (2009). Religious coping and caregiver well-being in Mexican-American families. Aging & Mental Health, 13(1), 84-91. https://doi.org/https://dx.doi.org/10.1080/13607860802154507

Hoshino, J., Hori, Y., Kondo, T., Tamakoshi, K., Toyoshima, H., & Sakakibara, H. (2013). Characteristics of hypertension-related factors in female home caregivers in Japan-comparison with general community non-caregivers. Journal of Clinical Nursing, 22(3-4), 466-478. https://doi.org/https://dx.doi.org/10.1111/j.1365-2702.2011.04039.x

Jansson, W., Grafstrom, M., & Winblad, B. (1997). Daughters and sons as caregivers for their demented and non-demented elderly parents. A part of a population-based study carried out in Sweden. Scandinavian Journal of Social Medicine, 25(4), 289-295. http://proxy.lib.umich.edu/login?url=https://search.ebscohost.com/login.aspx?direct=true&db=ccm&AN=107264636&site=ehost-live&scope=site

Jarrott, S. E., Zarit, S. H., Stephens, M. A. P., Townsend, A., & Greene, R. (2005). Instrumental help and caregivers' distress: Effects of change in informal and formal help. American journal of Alzheimer's disease and other dementias, 20(3), 181-190. https://doi.org/10.1177/153331750502000308

Jarrott, S. E., Zarit, S. H., Stephens, M. A. P., Townsend, A. L., & Greene, R. (2000). Effects of adult day service programs on time usage by employed and non-employed caregivers. Journal of Applied Gerontology, 19(4), 371-388. https://doi.org/10.1177/073346480001900401

Jenkins, T. S., Parham, I. A., & Jenkins, L. R. (1985). Alzheimer's disease: caregivers' perceptions of burden. Journal of Applied Gerontology, 4(2), 40-57. http://proxy.lib.umich.edu/login?url=https://search.ebscohost.com/login.aspx?direct=true&db=gnh&AN=40916&site=ehost-live&scope=site

Jensen, C. J., Ferrari, M., & Cavanaugh, J. C. (2004). Building on the benefits: assessing satisfaction and well-being in elder care. Ageing International, 29(1), 88-110. http://proxy.lib.umich.edu/login?url=https://search.ebscohost.com/login.aspx?direct=true&db=gnh&AN=104753&site=ehost-live&scope=site

Kosloski, K., Montgomery, R. J. V., & Youngbauer, J. G. (2001). Utilization of respite services: a comparison of users, seekers, and nonseekers. Journal of Applied Gerontology, 20(1), 111-132. http://proxy.lib.umich.edu/login?url=https://search.ebscohost.com/login.aspx?direct=true&db=gnh&AN=84208&site=ehost-live&scope=site

Kosloski, K., Young, R. F., & Montgomery, R. J. V. (1999). A new direction for intervention with depressed caregivers to Alzheimer's patients. Family Relations: An Interdisciplinary Journal of Applied Family Studies, 48(4), 373-379. https://doi.org/10.2307/585244

Kovaleva, M. A., Higgins, M., Dietrich, M. S., Jennings, B. M., Song, M.-K., Clevenger, C. K., Griffiths, P. C., & Hepburn, K. (2022). Characteristics associated with neuropsychiatric symptoms in persons living with dementia and caregiver distress and diminished well-being. Journal of the American Association of Nurse Practitioners, 34(4), 656-665. https://doi.org/https://dx.doi.org/10.1097/JXX.0000000000000681

Ku, L.-J. E., Chang, S.-M., Pai, M.-C., & Hsieh, H.-M. (2019). Predictors of caregiver burden and care costs for older persons with dementia in Taiwan. International Psychogeriatrics, 31(6), 885-894. https://doi.org/https://dx.doi.org/10.1017/S1041610218001382

Kumamoto, K., Arai, Y., & Zarit, S. H. (2006). Use of home care services effectively reduces feelings of burden among family caregivers of disabled elderly in Japan: Preliminary results. International Journal of Geriatric Psychiatry, 21(2), 163-170. https://doi.org/10.1002/gps.1445

Kumar, V., Ankuda, C. K., Aldridge, M. D., Husain, M., & Ornstein, K. A. (2020). Family Caregiving at the End of Life and Hospice Use: A National Study of Medicare Beneficiaries. Journal of the American Geriatrics Society, 68(10), 2288-2296. https://doi.org/https://dx.doi.org/10.1111/jgs.16648

Larkin, J. P., & Hopcroft, B. M. (1993). In-hospital respite as a moderator of caregiver stress. Health & Social Work, 18(2), 132-138. https://doi.org/10.1093/hsw/18.2.132

Lau, S., Chong, M. S., Ali, N., Chan, M., Chua, K. C., & Lim, W. S. (2015). Caregiver burden: Looking beyond the unidimensional total score. Alzheimer disease and associated disorders, 29(4), 338-346. https://doi.org/10.1097/WAD.0000000000000085

Lawton, M. P., Brody, E. M., Saperstein, A., & Grimes, M. (1989). Respite services for caregivers: research findings for service planning. Home health care services quarterly, 10(1-2), 5-32. http://proxy.lib.umich.edu/login?url=https://search.ebscohost.com/login.aspx?direct=true&db=gnh&AN=47699&site=ehost-live&scope=site

Lawton, M. P., Moss, M. S., Kleban, M. H., Glicksman, A., & Rovine, M. (1991). A two-factor model of caregiving appraisal and psychological well-being. Journal of Gerontology, 46(4), P181-P189. https://doi.org/10.1093/geronj/46.4.P181

Lee, D.-C. A., Burton, E., Slatyer, S., Jacinto, A., Oliveira, D., Bryant, C., Khushu, A., Tiller, E., Lalor, A., Watson, M., Layton, N., Brusco, N., & Hill, K. D. (2022). Understanding the Role, Quality of Life and Strategies Used by Older Carers of Older People to Maintain Their Own Health and Well-Being: A National Australian Survey. Clinical interventions in aging, 17, 1549-1567. https://doi.org/https://dx.doi.org/10.2147/CIA.S384202

Lee, H. S., Kim, D. K., & Kim, J.-H. (2006). Stress in caregivers of demented people in Korea--A modification of Pearlin and colleagues' stress model. International Journal of Geriatric Psychiatry, 21(8), 784-791. https://doi.org/10.1002/gps.1563

Lee, K., Yefimova, M., Puga, F., & Pickering, C. E. (2021). Gender Differences in Caregiver Burden Among Family Caregivers of Persons With Dementia. Journal of gerontological nursing, 47(7), 33-42. https://doi.org/https://dx.doi.org/10.3928/00989134-20210610-03

Lee, M., & Kolomer, S. (2005). Caregiver burden, dementia, and elder abuse in South Korea. Journal of elder abuse & neglect, 17(1), 61-74. https://doi.org/10.1300/J084v17n01_04

Lee, Y.-R., & Sung, K.-T. (1998). Cultural influences on caregiving burden: Cases of Koreans and Americans. The International Journal of Aging & Human Development, 46(2), 125-141. https://doi.org/10.2190/PM2C-V93R-NE8H-JWGV

Leggett, A. N., Meyer, O. L., Bugajski, B. C., & Polenick, C. A. (2021). Accentuate the positive: The association between informal and formal supports and caregiving gains. Journal of Applied Gerontology, 40(7), 763-771. https://doi.org/10.1177/0733464820914481

Leggett, A. N., Polenick, C. A., Maust, D. T., & Kales, H. C. (2018). Falls and Hospitalizations Among Persons With Dementia and Associated Caregiver Emotional Difficulties. The Gerontologist, 58(2), e78-e86. https://doi.org/https://dx.doi.org/10.1093/geront/gnx202

Lei, L., Maust, D. T., & Leggett, A. N. (2023). Functional Decline Over Time and Change in Family and Other Unpaid Care Provided to Community-Dwelling Older Adults Living With and Without Dementia. Journals of Gerontology Series B: Psychological Sciences & Social Sciences, 78(10), 1727-1734. https://doi.org/10.1093/geronb/gbad107

Lévesque, L., Cossette, S., & Lachance, L. (1998). Predictors of the psychological well-being of primary caregivers living with a demented relative: A 1-year follow-up study. Journal of Applied Gerontology, 17(2), 240-258. https://doi.org/10.1177/073346489801700211

Lévesque, L., Cossette, S., & Laurin, L. (1995). A multidimensional examination of the psychological and social well-being of caregivers of a demented relative. Research on aging, 17(3), 332-360. https://doi.org/10.1177/0164027595173005

Levesque, L., Cossette, S., Potvin, L., & Benigeri, M. (2000). Community services and caregivers of a demented relative: users and those perceiving a barrier to their use. Canadian Journal on Aging, 19(2), 186-209. http://proxy.lib.umich.edu/login?url=https://search.ebscohost.com/login.aspx?direct=true&db=gnh&AN=82164&site=ehost-live&scope=site

Liang, J., Aranda, M. P., & Lloyd, D. A. (2020). Association between role overload and sleep disturbance among dementia caregivers: The impact of social support and social engagement. Journal of aging and health, 32(10), 1345-1354. https://doi.org/10.1177/0898264320926062

Lieberman, M. A., & Kramer, J. H. (1991). Factors affecting decisions to institutionalize demented elderly. Gerontologist, 31(3), 371-374. http://proxy.lib.umich.edu/login?url=https://search.ebscohost.com/login.aspx?direct=true&db=gnh&AN=52195&site=ehost-live&scope=site

Liu, H., Fang, B., Chan, J., & Chen, G. (2019). The relationship between comorbidities in dementia patients and burden on adult–child primary caregivers: Does having a secondary caregiver matter? International journal of mental health nursing, 28(6), 1306-1317. https://doi.org/10.1111/inm.12640

Liu, Y., Song, Y., Johnson, F. U., Lei, L., Choi, S.-W. E., Antonucci, T. C., & Robinson-Lane, S. G. (2023). Characteristics and Predictors of Sleep Among Spousal Care Dyads Living With Chronic Conditions. The journals of gerontology. Series B, Psychological sciences and social sciences, 78(Suppl 1), S38-S47. https://doi.org/https://dx.doi.org/10.1093/geronb/gbac096

Lowenstein, A. (1999). Caring for parents with Alzheimer's: Comparing perceptions of physical and mental health in the Jewish and Arab sectors in Israel. Journal of cross-cultural gerontology, 14(1), 65-76. https://doi.org/10.1023/A:1006654621517

Majerovitz, S. D. (2001). Formal versus informal support: Stress buffering among dementia caregivers. Journal of Mental Health and Aging, 7(4), 413-423. http://proxy.lib.umich.edu/login?url=https://search.ebscohost.com/login.aspx?direct=true&db=psyh&AN=2002-00606-003&site=ehost-live&scope=site

Malak, R. E. Z., Krawczyk-Wasielewska, A., Glodowska, K., Grobelny, B., Kleka, P., Mojs, E., Keczmer, P., & Samborski, W. (2016). Condition of informal caregivers in long-term care of people with dementi. Annals of agricultural and environmental medicine : AAEM, 23(3), 491-494. https://doi.org/https://dx.doi.org/10.5604/12321966.1219193

Martichuski, D. K., Knight, B. L., Karlin, N. J., & Bell, P. A. (1997). Correlates of Alzheimer's disease caregivers' support group attendance. Activities, Adaptation & Aging, 21(4), 27-40. https://doi.org/10.1300/j016v21n04_04

McLoughlin, C., Goranitis, I., & Al-Janabi, H. (2020). Validity and Responsiveness of Preference-Based Quality-of-Life Measures in Informal Carers: A Comparison of 5 Measures Across 4 Conditions. Value in health : the journal of the International Society for Pharmacoeconomics and Outcomes Research, 23(6), 782-790. https://doi.org/https://dx.doi.org/10.1016/j.jval.2020.01.015

Messina, A., Lattanzi, M., Albanese, E., & Fiordelli, M. (2022). Caregivers of people with dementia and mental health during COVID-19: findings from a cross-sectional study. BMC Geriatrics, 22(1), 56. https://doi.org/https://dx.doi.org/10.1186/s12877-022-02752-x

Meyer, O. L., Koo, H. J., Strominger, J., Tran, D., Bach, A., & Leggett, A. N. (2022). Neighborhood Characteristics and Caregiver Depressive Symptoms in the National Study of Caregiving. Journal of Aging & Health, 34(6/8), 1005-1015. https://doi.org/10.1177/08982643221085106

Mills, P. J., Adler, K. A., Dimsdale, J. E., Perez, C. J., Ziegler, M. G., Ancoli-Israel, S., Patterson, T. L., & Grant, I. (2004). Vulnerable caregivers of Alzheimer disease patients have a deficit in β₂-adrenergic receptor sensitivity and density. The American Journal of Geriatric Psychiatry, 12(3), 281-286. https://doi.org/10.1176/appi.ajgp.12.3.281

Mittelman, M. S. (2000). Effect of support and counseling on caregivers of patients with Alzheimer's disease. International Psychogeriatrics, 12(Suppl1), 341-346. https://doi.org/10.1017/S1041610200007250

Mittelman, M. S., Ferris, S. H., Shulman, E., Steinberg, G., Ambinder, A., Mackell, J. A., & Cohen, J. (1995). A comprehensive support program: Effect on depression in spouse-caregivers of AD patients. The Gerontologist, 35(6), 792-802. https://doi.org/10.1093/geront/35.6.792

Mittelman, M. S., Ferris, S. H., Steinberg, G., Shulman, E., Mackell, J. A., Ambinder, A., & Cohen, J. (1993). An intervention that delays institutionalization of Alzheimer's disease patients: Treatment of spouse-caregivers. The Gerontologist, 33(6), 730-740. https://doi.org/10.1093/geront/33.6.730

Miyawaki, C. E., McClellan, A., Bouldin, E. D., Brohard, C., Spencer, H., Tahija, N., & Kunik, M. E. (2023). Feasibility and Efficacy of Life Review Delivered by Virtually-Trained Family Caregivers. Journal of Alzheimer's disease : JAD, 95(2), 573-583. https://doi.org/https://dx.doi.org/10.3233/JAD-230371

Monahan, D. J., Greene, V. L., & Coleman, P. D. (1992). Caregiver support groups: Factors affecting use of services. Social Work, 37(3), 254-260. http://proxy.lib.umich.edu/login?url=https://search.ebscohost.com/login.aspx?direct=true&db=psyh&AN=1992-40303-001&site=ehost-live&scope=site

Monahan, D. J., & Hooker, K. (1995). Health of spouse caregivers of dementia patients: the role of personality and social support. Social Work, 40(3), 305-314. http://proxy.lib.umich.edu/login?url=https://search.ebscohost.com/login.aspx?direct=true&db=gnh&AN=63421&site=ehost-live&scope=site

Monohan, D. J. (1993). Utilization of dementia-specific respite day care for clients and their caregivers in a social model program. Journal of Gerontological Social Work, 20(3-4), 57-70. http://proxy.lib.umich.edu/login?url=https://search.ebscohost.com/login.aspx?direct=true&db=gnh&AN=60075&site=ehost-live&scope=site

Montorio, I., Losada, A., Izal, M., & Marquez, M. (2009). Dysfunctional thoughts about caregiving questionnaire: psychometric properties of a new measure. International Psychogeriatrics, 21(5), 913-921. https://doi.org/https://dx.doi.org/10.1017/S1041610209990366

Moon, H., & Dilworth-Anderson, P. (2015). Baby boomer caregiver and dementia caregiving: findings from the National Study of Caregiving. Age and ageing, 44(2), 300-306. https://doi.org/https://dx.doi.org/10.1093/ageing/afu119

Morimoto, H., & Takebayashi, Y. (2021). Antecedents and Outcomes of Enrichment Among Working Family Caregivers of People With Dementia: A Longitudinal Analysis. The journals of gerontology. Series B, Psychological sciences and social sciences, 76(6), 1060-1070. https://doi.org/https://dx.doi.org/10.1093/geronb/gbaa183

Neubauer, S., Holle, R., Menn, P., & Gräßel, E. (2009). A valid instrument for measuring informal care time for people with dementia. International Journal of Geriatric Psychiatry, 24(3), 275-282. http://proxy.lib.umich.edu/login?url=https://search.ebscohost.com/login.aspx?direct=true&db=gnh&AN=EP36555222&site=ehost-live&scope=site

Nikzad-Terhune, K. A., Anderson, K. A., Newcomer, R., & Gaugler, J. E. (2010). Do trajectories of at-home dementia caregiving account for burden after nursing home placement? A growth curve analysis. Social work in health care, 49(8), 734-752. https://doi.org/10.1080/00981381003635296

Nobili, A., Riva, E., Tettamanti, M., Lucca, U., Liscio, M., Petrucci, B., & Porro, G. S. (2004). The effect of a structured intervention on caregivers of patients with dementia and problem behaviors: A randomized controlled pilot study. Alzheimer disease and associated disorders, 18(2), 75-82. https://doi.org/10.1097/01.wad.0000126618.98867.fc

Ondee, P., Panitrat, R., Pongthavornkamol, K., Senanarong, V., Harvath, T. A., & Nittayasudhi, D. (2013). Factors prediscting depression among caregivers of persons with dementia. Pacific Rim International Journal of Nursing Research, 17(2), 167-180.

Onishi, J., Suzuki, Y., Umegaki, H., Nakamura, A., Endo, H., & Iguchi, A. (2005). Influence of behavioral and psychological symptoms of dementia (BPSD) and environment of care on caregivers' burden. Archives of gerontology and geriatrics, 41(2), 159-168. https://doi.org/10.1016/j.archger.2005.01.004

Orgeta, V., Orrell, M., Hounsome, B., Woods, B., & Remcare, t. (2015). Self and carer perspectives of quality of life in dementia using the QoL-AD. International Journal of Geriatric Psychiatry, 30(1), 97-104. https://doi.org/https://dx.doi.org/10.1002/gps.4130

Ornstein, K. A., Wolff, J. L., Bollens-Lund, E., Rahman, O.-K., & Kelley, A. S. (2019). Spousal Caregivers Are Caregiving Alone In The Last Years Of Life. Health affairs (Project Hope), 38(6), 964-972. https://doi.org/https://dx.doi.org/10.1377/hlthaff.2019.00087

Osaki, T., Morikawa, T., Kajita, H., Kobayashi, N., Kondo, K., & Maeda, K. (2016). Caregiver burden and fatigue in caregivers of people with dementia: Measuring human herpesvirus (HHV)-6 and -7 DNA levels in saliva. Archives of gerontology and geriatrics, 66, 42-48. https://doi.org/10.1016/j.archger.2016.04.015

Park, D., Morano, C., & Savage, A. (2021). Understanding the role of social support and social support network for depression among informal dementia caregivers: a pilot clinical project on caregivers in NYC. Social work in health care, 60(8-9), 599-613. https://doi.org/https://dx.doi.org/10.1080/00981389.2021.1987374

Park, M., Choi, S., Lee, S. J., Kim, S. H., Kim, J., Go, Y., & Lee, D. Y. (2018). The roles of unmet needs and formal support in the caregiving satisfaction and caregiving burden of family caregivers for persons with dementia. International Psychogeriatrics, 30(4), 557-567. https://doi.org/10.1017/S104161021700196X

Patterson, S. E., Tate, A. M., Hu, Y.-L., Wang, J., Schoeni, R. F., & Choi, H. (2023). The Social Cost of Providing Care to Older Adults With and Without Dementia. Journals of Gerontology Series B: Psychological Sciences & Social Sciences, 78, S71-S80. https://doi.org/10.1093/geronb/gbac146

Peng, L.-M., Chiu, Y.-C., Liang, J., & Chang, T. H. (2018). Risky wandering behaviors of persons with dementia predict family caregivers' health outcomes. Aging & Mental Health, 22(12), 1650-1657. https://doi.org/10.1080/13607863.2017.1387764

Penning, M. J. (1995). Cognitive impairment, caregiver burden, and the utilization of home health services. Journal of aging and health, 7(2), 233-253. http://proxy.lib.umich.edu/login?url=https://search.ebscohost.com/login.aspx?direct=true&db=gnh&AN=63300&site=ehost-live&scope=site

Perodeau, G., Lauzon, S., Levesque, L., & Lachance, L. (2001). Mental health, stress correlates and psychotropic drug use or non-use among aged caregivers to elders with dementia. Aging & Mental Health, 5(3), 225-234. http://ovidsp.ovid.com/ovidweb.cgi?T=JS&PAGE=reference&D=med4&NEWS=N&AN=11575061

Pett, M. A., Caserta, M. S., Hutton, A. P., & Lund, D. A. (1988). Intergenerational conflict: Middle-aged women caring for demented older relatives. American Journal of Orthopsychiatry, 58(3), 405-417. https://doi.org/10.1111/j.1939-0025.1988.tb01601.x

Pierce, L., Ader, K., & Peter, P. (1989). Caregiver burden and coping strategies. American Journal of Alzheimer's Care and Related Disorders and Research, 4(5), 36-41. http://proxy.lib.umich.edu/login?url=https://search.ebscohost.com/login.aspx?direct=true&db=gnh&AN=47992&site=ehost-live&scope=site

Pillemer, K., & Suitor, J. J. (1996). 'It takes one to help one': Effects of similar others on the well-being of caregivers. The Journals of Gerontology: Series B: Psychological Sciences and Social Sciences, 51(5), S250-S257. https://doi.org/10.1093/geronb/51B.5.S250

Pinto, M. F., Barbosa, D. A., Ferreti, C. E. L., de Souza, L. F., Fram, D. S., & Belasco, A. G. S. (2009). Quality of life among caregivers of elders with Alzheimer's disease. Acta Paulista de Enfermagem, 22(5), 652-657. http://proxy.lib.umich.edu/login?url=https://search.ebscohost.com/login.aspx?direct=true&db=ccm&AN=105261927&site=ehost-live&scope=site

Pot, A. M., Zarit, S. H., Twisk, J. W. R., & Townsend, A. L. (2005). Transitions in caregivers' use of paid home help: associations with stress appraisals and well-being. Psychology and aging, 20(2), 211-219. http://ovidsp.ovid.com/ovidweb.cgi?T=JS&PAGE=reference&D=med6&NEWS=N&AN=16029085

Price, H. J., & Levy, K. A. (1990). Variables influencing burden in spousal and adult child primary caregivers of persons with Alzheimer's disease in the home setting. American Journal of Alzheimer's Care and Related Disorders and Research, 5(1), 34-42. http://proxy.lib.umich.edu/login?url=https://search.ebscohost.com/login.aspx?direct=true&db=gnh&AN=48431&site=ehost-live&scope=site

Pruchno, R. A. (1990). The effects of help patterns on the mental health of spouse caregivers. Research on aging, 12(1), 57-71. https://doi.org/10.1177/0164027590121003

Pruchno, R. A., & Resch, N. L. (1989). Husbands and wives as caregivers: Antecedents of depression and burden. The Gerontologist, 29(2), 159-165. https://doi.org/10.1093/geront/29.2.159

Puga, F., Wang, D., Rafford, M., Poe, A., & Pickering, C. E. Z. (2023). The relationship between daily stressors, social support, depression and anxiety among dementia family caregivers: a micro-longitudinal study. Aging & Mental Health, 27(7), 1291-1299. https://doi.org/10.1080/13607863.2022.2116392

Raina, P., McLntyre, C., Zhu, B., McDowell, I., Santaguida, L., Kristjansson, B., Hendricks, A., Massfeller, H., & Chambers, L. W. (2004). Understanding the Influence of the Complex Relationships among Informal and Formal Supports on the Well-Being of Caregivers of Persons with Dementia. Canadian Journal on Aging, 23(Suppl1), S49-S59. http://proxy.lib.umich.edu/login?url=https://search.ebscohost.com/login.aspx?direct=true&db=psyh&AN=2004-22386-004&site=ehost-live&scope=site

Riffin, C., Van Ness, P. H., Wolff, J. L., & Fried, T. (2019). Multifactorial examination of caregiver burden in a national sample of family and unpaid caregivers. Journal of the American Geriatrics Society, 67(2), 277-283. https://doi.org/10.1111/jgs.15664

Roberto, K. A., & Savla, J. (2022). Extended Family Caregivers for Persons Living With Dementia. Journal of family nursing, 28(4), 396-407. https://doi.org/10.1177/10748407221115455

Robinson, K., & Austin, J. K. (1998). Wife caregivers' and supportive others' perceptions of the caregivers' health and social support. Research in Nursing & Health, 21(1), 51-57. http://proxy.lib.umich.edu/login?url=https://search.ebscohost.com/login.aspx?direct=true&db=ccm&AN=107263351&site=ehost-live&scope=site

Rodriguez, G., De Leo, C., Girtler, N., Vitali, P., Grossi, E., & Nobili, F. (2003). Psychological and social aspects in management of Alzheimer's patients: an inquiry among caregivers. Neurological sciences : official journal of the Italian Neurological Society and of the Italian Society of Clinical Neurophysiology, 24(5), 329-335. http://ovidsp.ovid.com/ovidweb.cgi?T=JS&PAGE=reference&D=med5&NEWS=N&AN=14716528

Roland, K. P., & Chappell, N. L. (2019). Caregiver Experiences Across Three Neurodegenerative Diseases: Alzheimer's, Parkinson's, and Parkinson's With Dementia. Journal of aging and health, 31(2), 256-279. https://doi.org/https://dx.doi.org/10.1177/0898264317729980

Rosdinom, R., Zarina, M. Z. N., Zanariah, M. S., Marhani, M., & Suzaily, W. (2013). Behavioural and psychological symptoms of dementia, cognitive impairment and caregiver burden in patients with dementia. Preventive medicine, 57 Suppl, S67-69. https://doi.org/https://dx.doi.org/10.1016/j.ypmed.2012.12.025

Roth, D. L., Mittelman, M. S., Clay, O. J., Madan, A., & Haley, W. E. (2005). Changes in social support as mediators of the impact of a psychosocial intervention for spouse caregivers of persons with Alzheimer's disease. Psychology and aging, 20(4), 634-644. http://proxy.lib.umich.edu/login?url=https://search.ebscohost.com/login.aspx?direct=true&db=gnh&AN=111040&site=ehost-live&scope=site

Sakka, M., Goto, J., Kita, S., Sato, I., Soejima, T., & Kamibeppu, K. (2019). Associations among behavioral and psychological symptoms of dementia, care burden, and family‐to‐work conflict of employed family caregivers. Geriatrics & gerontology international, 19(1), 51-55. https://doi.org/10.1111/ggi.13556

Salguero, R. H., Kohn, R., Salguero, L. F., & Marotta, C. A. (1998). Caregivers of persons with Alzheimer's disease: Cultural differences in perceived caregiver burden in Guatemala and Rhode Island. Journal of cross-cultural gerontology, 13(3), 229-240. https://doi.org/10.1023/A:1006534521253

Schlag, K. E., & Vangelisti, A. L. (2023). Reflections on dementia-related stigma and direct support seeking by family caregivers as mediating associations between caregiver stress, burden, and well-being. Health communication. https://doi.org/10.1080/10410236.2023.2270248

Schmidt, A., Ayoub, M. F., de Souza, Y. L. P., Guimarães, A. T. B., & Foss, M. P. (2021). COVID-19 pandemic and mental health of a sample of Brazilian caregivers of people with dementia. Dementia & neuropsychologia, 15(4), 448-457. https://doi.org/10.1590/1980-57642021dn15-040004

Schulz, R., Belle, S. H., Czaja, S. J., McGinnis, K. A., Stevens, A., & Zhang, S. (2004). Long-term care placement of dementia patients and caregiver health and well-being. JAMA, 292(8), 961-967. http://ovidsp.ovid.com/ovidweb.cgi?T=JS&PAGE=reference&D=med5&NEWS=N&AN=15328328

Schulz, R., & Williamson, G. M. (1991). 2-year longitudinal study of depression among Alzheimer's caregivers. Psychology and aging, 6(4), 569-578. http://proxy.lib.umich.edu/login?url=https://search.ebscohost.com/login.aspx?direct=true&db=gnh&AN=53615&site=ehost-live&scope=site

Shankar, K. N., Hirschman, K. B., Hanlon, A. L., & Naylor, M. D. (2014). Burden in caregivers of cognitively impaired elderly adults at time of hospitalization: A cross‐sectional analysis. Journal of the American Geriatrics Society, 62(2), 276-284. https://doi.org/10.1111/jgs.12657

Shanks-McElroy, H. A., & Strobino, J. (2001). Male caregivers of spouses with Alzheimer's disease: Risk factors and health status. American Journal of Alzheimer's Disease, 16(3), 167-175. https://doi.org/10.1177/153331750101600308

Shikiar, R., Shakespeare, A., Sagnier, P.-P., Wilkinson, D., McKeith, I., Dartigues, J.-F., & Dubois, B. (2000). The impact of metrifonate therapy on caregivers of patients with Alzheimer's disease: Results from the MALT clinical trial. Journal of the American Geriatrics Society, 48(3), 268-274. https://doi.org/10.1111/j.1532-5415.2000.tb02645.x

Shikimoto, R., Sado, M., Ninomiya, A., Yoshimura, K., Ikeda, B., Baba, T., & Mimura, M. (2018). Predictive factors associated with psychological distress of caregivers of people with dementia in Japan: a cross-sectional study. International Psychogeriatrics, 30(8), 1089-1098. https://doi.org/https://dx.doi.org/10.1017/S1041610217002289

Smyth, K. A., & Milidonis, M. K. (1999). The relationship between normative beliefs about help seeking and the experience of caregiving in Alzheimer's disease. Journal of Applied Gerontology, 18(2), 222-238. https://doi.org/10.1177/073346489901800206

Song, M.-K., Paul, S., Happ, M. B., Lea, J., Pirkle, J. L., Jr., & Turberville-Trujillo, L. (2023). Informal Caregiving Networks of Older Adults With Dementia Superimposed on Multimorbidity: A Social Network Analysis Study. Innovation in aging, 7(4), igad033. https://doi.org/https://dx.doi.org/10.1093/geroni/igad033

Spruytte, N., Van Audenhove, C., & Lammertyn, F. (2001). Predictors of institutionalization of cognitively-impaired elderly cared for by their relatives. International Journal of Geriatric Psychiatry, 16(12), 1119-1128. http://proxy.lib.umich.edu/login?url=https://search.ebscohost.com/login.aspx?direct=true&db=gnh&AN=88033&site=ehost-live&scope=site

Steffen, A. M., Thompson, L. W., Gallagher-Thompson, D., & Koin, D. (1999). Physical and psychosocial correlates of hormone replacement therapy with chronically stressed postmenopausal women. Journal of aging and health, 11(1), 3-26. https://doi.org/10.1177/089826439901100101

Stommel, M., Collins, C. E., & Given, B. A. (1994). Costs of family contributions to the care of persons with dementia. Gerontologist, 34(2), 199-205. http://proxy.lib.umich.edu/login?url=https://search.ebscohost.com/login.aspx?direct=true&db=gnh&AN=60614&site=ehost-live&scope=site

Suitor, J. J., & Pillemer, K. (1993). Support and interpersonal stress in the social networks of married daughters caring for parents with dementia. Journals of Gerontology, 48(1), S1-S8. http://proxy.lib.umich.edu/login?url=https://search.ebscohost.com/login.aspx?direct=true&db=gnh&AN=57180&site=ehost-live&scope=site

Suitor, J. J., & Pillemer, K. (1994). Family caregiving and marital satisfaction: Findings from a 1-year panel study of women caring for parents with dementia. Journal of Marriage and the Family, 56(3), 681-690. https://doi.org/10.2307/352878

Suitor, J. J., & Pillemer, K. (1996). Sources of support and interpersonal stress in the networks of married caregiving daughters: findings from a 2-year longitudinal study. Journals of Gerontology Series B: Psychological Sciences & Social Sciences, 51B(6), S297-306. https://doi.org/10.1093/geronb/51b.6.s297

Sutcliffe, C., Giebel, C., Bleijlevens, M., Lethin, C., Stolt, M., Saks, K., Soto, M. E., Meyer, G., Zabalegui, A., Chester, H., & Challis, D. (2017). Caring for a Person With Dementia on the Margins of Long-Term Care: A Perspective on Burden From 8 European Countries. Journal of the American Medical Directors Association, 18(11), 967-973.e961. https://doi.org/10.1016/j.jamda.2017.06.004

Sutcliffe, C. L., Giebel, C. M., Jolley, D., & Challis, D. J. (2016). Experience of burden in carers of people with dementia on the margins of long‐term care. International Journal of Geriatric Psychiatry, 31(2), 101-108. https://doi.org/10.1002/gps.4295

Takahashi, M., Tanaka, K., & Miyaoka, H. (2005). Depression and associated factors of informal caregivers versus professional caregivers of demented patients. Psychiatry and clinical neurosciences, 59(4), 473-480. http://ovidsp.ovid.com/ovidweb.cgi?T=JS&PAGE=reference&D=med6&NEWS=N&AN=16048454

Tan, K. P., Ang, J. K., Koh, E. B. Y., Pang, N. T. P., & Mat Saher, Z. (2023). Relationship of Psychological Flexibility and Mindfulness to Caregiver Burden, and Depressive and Anxiety Symptoms in Caregivers of People with Dementia. International journal of environmental research and public health, 20(5). https://doi.org/https://dx.doi.org/10.3390/ijerph20054232

Terayama, H., Sakurai, H., Namioka, N., Jaime, R., Otakeguchi, K., Fukasawa, R., Sato, T., Hirao, K., Kanetaka, H., Shimizu, S., Umahara, T., & Hanyu, H. (2018). Caregivers' education decreases depression symptoms and burden in caregivers of patients with dementia. Psychogeriatrics : the official journal of the Japanese Psychogeriatric Society, 18(5), 327-333. https://doi.org/https://dx.doi.org/10.1111/psyg.12337

Thomas, P., Chantoin-Merlet, S., Hazif-Thomas, C., Belmin, J., Montagne, B., Clément, J.-P., Lebruchec, M., & Billon, R. (2002). Complaints of informal caregivers providing home care for dementia patients: The Pixel study. International Journal of Geriatric Psychiatry, 17(11), 1034-1044. https://doi.org/10.1002/gps.746

Tomita, M. R., Sarang, A., Lee, K., Lee, K. S., Russ, L. S., & Noe, M. (2010). Characteristics and perceived supports of primary caregivers of home-based older adults with dementia in India, Taiwan, and the United States. Topics in Geriatric Rehabilitation, 26(1), 2-16. https://doi.org/10.1097/TGR.0b013e3181cd69ac

Tommis, Y., Seddon, D., Woods, B., Robinson, C. A., Reeves, C., & Russell, I. T. (2007). Rural-urban differences in the effects on mental well-being of caring for people with stroke or dementia. Aging & Mental Health, 11(6), 743-750. https://doi.org/10.1080/13607860701365972

Vaingankar, J. A., Chong, S. A., Abdin, E., Picco, L., Jeyagurunathan, A., Zhang, Y., Sambasivam, R., Chua, B. Y., Ng, L. L., Prince, M., & Subramaniam, M. (2016). Care participation and burden among informal caregivers of older adults with care needs and associations with dementia. International Psychogeriatrics, 28(2), 221-231. https://doi.org/https://dx.doi.org/10.1017/S104161021500160X

Vaingankar, J. A., Chong, S. A., Abdin, E., Picco, L., Shafie, S., Seow, E., Pang, S., Sagayadevan, V., Chua, B. Y., Chua, H. C., & Subramaniam, M. (2016). Psychiatric morbidity and its correlates among informal caregivers of older adults. Comprehensive psychiatry, 68, 178-185. https://doi.org/https://dx.doi.org/10.1016/j.comppsych.2016.04.017

Valimaki, T. H., Vehvilainen-Julkunen, K. M., Pietila, A.-M. K., & Pirttila, T. A. (2009). Caregiver depression is associated with a low sense of coherence and health-related quality of life. Aging & Mental Health, 13(6), 799-807. https://doi.org/https://dx.doi.org/10.1080/13607860903046487

van der Heide, I., van Wezel, N., Blom, M., Spreeuwenberg, P., Deville, W. L. J. M., & Francke, A. L. (2021). Effects of an educational intervention on health-related quality of life among family caregivers of people with dementia with a Turkish or Moroccan immigrant background: Insights from a cluster randomised controlled trial. Patient education and counseling, 104(5), 1168-1175. https://doi.org/https://dx.doi.org/10.1016/j.pec.2020.10.029

Vernooij-Dassen, M., Felling, A., & Persoon, J. (1997). Predictors of change and continuity in home care for dementia patients. International Journal of Geriatric Psychiatry, 12(6), 671-677. https://doi.org/10.1002/(SICI)1099-1166(199706)12:6<671::AID-GPS599>3.0.CO;2-G

Vidoni, E. D., Perales, J., Alshehri, M., Giles, A.-M., Siengsukon, C. F., & Burns, J. M. (2019). Aerobic Exercise Sustains Performance of Instrumental Activities of Daily Living in Early-Stage Alzheimer Disease. Journal of Geriatric Physical Therapy, 42(3), E129-E134. https://doi.org/10.1519/JPT.0000000000000172

Wang, P.-C., Yip, P.-K., & Chang, Y. (2016). Self-efficacy and sleep quality as mediators of perceived stress and memory and behavior problems in the link to dementia caregivers' depression in Taiwan. Clinical Gerontologist: The Journal of Aging and Mental Health, 39(3), 222-239. https://doi.org/10.1080/07317115.2015.1128503

Wang, Y.-N., Hsu, W.-C., Yang, P.-S., Yao, G., Chiu, Y.-C., Chen, S.-T., Huang, T.-H., & Shyu, Y.-I. L. (2018). Caregiving demands, job demands, and health outcomes for employed family caregivers of older adults with dementia: Structural equation modeling. Geriatric Nursing, 39(6), 676-682. https://doi.org/10.1016/j.gerinurse.2018.05.003

Warrington, J., & Eagles, J. M. (1996). A comparison of cognitively impaired attenders and their coresident carers at day hospitals and day centres in aberdeen. International Journal of Geriatric Psychiatry, 11(3), 251-256. https://doi.org/10.1002/(SICI)1099-1166(199603)11:3<251::AID-GPS351>3.0.CO;2-6

Wiegelmann, H., Wolf-Ostermann, K., Brannath, W., Arzideh, F., Dreyer, J., Thyrian, R., Schirra-Weirich, L., & Verhaert, L. (2021). Sociodemographic aspects and health care-related outcomes: a latent class analysis of informal dementia care dyads. BMC Health Services Research, 21(1), 727. https://doi.org/https://dx.doi.org/10.1186/s12913-021-06708-6

Wiegelmann, H., Wolf-Ostermann, K., Janssen, N., van Hout, H., Vroomen, J. L. M., & Arzideh, F. (2023). Sociodemographic structure and health care-related outcomes of community-dwelling dementia caregiving dyads: a latent class replication study. BMC Health Services Research, 23(1), 482. https://doi.org/https://dx.doi.org/10.1186/s12913-023-09505-5

Wojtyna, E., & Popiołek, K. (2012). Character of the relationship with Alzheimer patient and the psychological costs of care. Polish Psychological Bulletin, 43(4), 244-252. https://doi.org/10.2478/v10059-012-0027-0

Wood, J. B., & Parham, I. A. (1990). Coping with perceived burden: ethnic and cultural issues in Alzheimer's family caregiving. Journal of Applied Gerontology, 9(3), 325-339. http://proxy.lib.umich.edu/login?url=https://search.ebscohost.com/login.aspx?direct=true&db=gnh&AN=50388&site=ehost-live&scope=site

Wulff, J., Fange, A. M., Lethin, C., & Chiatti, C. (2020). Self-reported symptoms of depression and anxiety among informal caregivers of persons with dementia: a cross-sectional comparative study between Sweden and Italy. BMC Health Services Research, 20(1), 1114. https://doi.org/https://dx.doi.org/10.1186/s12913-020-05964-2

Yordi, C., DuNah, R., Bostrom, A., Fox, P., Wilkinson, A., & Newcomer, R. (1997). Caregiver supports: outcomes from the Medicare Alzheimer's Disease Demonstration. Health Care Financing Review, 19(2), 97-117. http://proxy.lib.umich.edu/login?url=https://search.ebscohost.com/login.aspx?direct=true&db=gnh&AN=74745&site=ehost-live&scope=site

Youn, G., Knight, B. G., Jeong, H.-S., & Benton, D. (1999). Differences in familism values and caregiving outcomes among Korean, Korean American, and White American dementia caregivers. Psychology and aging, 14(3), 355-364. https://doi.org/10.1037/0882-7974.14.3.355

Young, R. F. (2003). Nursing home admission of female Alzheimer's patients: family care aspects. Women's health issues : official publication of the Jacobs Institute of Women's Health, 13(1), 2-7. http://ovidsp.ovid.com/ovidweb.cgi?T=JS&PAGE=reference&D=med5&NEWS=N&AN=12598053

Zarit, S. H., Anthony, C. R., & Boutselis, M. (1987). Interventions with caregivers of dementia patients: comparison of two approaches. Psychology and aging, 2(3), 225-232. http://proxy.lib.umich.edu/login?url=https://search.ebscohost.com/login.aspx?direct=true&db=gnh&AN=41546&site=ehost-live&scope=site

Zucca, M., Isella, V., Lorenzo, R. D., Marra, C., Cagnin, A., Cupidi, C., Bonanni, L., Laganà, V., Rubino, E., Vanacore, N., Agosta, F., Caffarra, P., Sambati, R., Quaranta, D., Guglielmi, V., Appollonio, I. M., Logroscino, G., Filippi, M., Tedeschi, G., . . . Bruni, A. C. (2021). Being the family caregiver of a patient with dementia during the Coronavirus disease 2019 lockdown. Frontiers in aging neuroscience, 13. https://doi.org/10.3389/fnagi.2021.653533
